# A Short Indel-Lacking-Resistance Gene Triggers Silencing of the Photosynthetic Machinery Components Through TYLCSV-Associated Endogenous siRNAs in Tomato

**DOI:** 10.3389/fpls.2018.01470

**Published:** 2018-10-11

**Authors:** Michela Chiumenti, Claudia Rita Catacchio, Laura Miozzi, Walter Pirovano, Mario Ventura, Vitantonio Pantaleo

**Affiliations:** ^1^Institute for Sustainable Plant Protection of the National Research Council, Research Unit of Bari, Bari, Italy; ^2^Dipartimento di Biologia, Università degli Studi di Bari Aldo Moro, Bari, Italy; ^3^Institute for Sustainable Plant Protection of the National Research Council, Research Unit of Turin, Turin, Italy; ^4^BaseClear B.V., Leiden, Netherlands

**Keywords:** functional indels, miRNA-mediated regulation, secondary siRNAs, viral symptoms, NBS-LRR clade

## Abstract

Plant viruses modify gene expression in infected tissues by altering the micro (mi)RNA-mediated regulation of genes. Among conserved miRNA targets there are transcripts coding for transcription factors, RNA silencing core, and disease-resistance proteins. Paralogs in these gene families are widely present in plant genomes and are known to respond differently to miRNA-mediated regulation during plant virus infections. Using genome-wide approaches applied to *Solanum lycopersicum* infected by a nuclear-replicating virus, we highlighted miRNA-mediated cleavage events that could not be revealed in virus-free systems. Among them we confirmed miR6024 targeting and cleavage of RX-coiled-coil (RX-CC), nucleotide binding site (NBS), leucine-rich (LRR) mRNA. Cleavage of paralogs was associated with short indels close to the target sites, indicating a general functional significance of indels in fine-tuning gene expression in plant–virus interaction. miR6024-mediated cleavage, uniquely in virus-infected tissues, triggers the production of several 21–22 nt secondary siRNAs. These secondary siRNAs, rather than being involved in the cascade regulation of other NBS–LRR paralogs, explained cleavages of several mRNAs annotated as defence-related proteins and components of the photosynthetic machinery. Outputs of these data explain part of the phenotype plasticity in plants, including the appearance of yellowing symptoms in the viral pathosystem.

## Introduction

RNA silencing refers to conserved pathways affecting gene expression through negative regulation mediated by non-coding RNAs, such as short interfering (si)RNAs. In plants, micro (mi)RNA comprises one of the most abundant classes of 21- to 24-nucleotide (nt)-long small RNAs that mediate post-transcriptional regulation of endogenous messenger (m)RNAs (Bartel, 2004). The biogenesis and activity of plant miRNAs are regulated by DICER-LIKE1 (DCL1) and Argonaute1 (AGO1), respectively. miRNAs function as a guide by base-pairing with their target RNAs, whereas AGO1 [mainly, but not exclusively (Adenot et al., 2006)] plays a role as effector, recruiting factors that induce mRNA translational repression and/or mRNA cleavage (Bartel, 2004; Voinnet, 2009; Iwakawa and Tomari, 2013). The 3′- and 5′-cleavage remnants of targeted mRNAs can be detected either by Northern blot analysis or rapid amplification of cDNA ends (RACE). The 5′-uncapped fragments detected by 5′RACE correspond to cleavage between the 10th and 11th nucleotides of miRNA/target site pairs. A genome-wide 5′-RACE analysis using next-generation sequencing, commonly known as parallel analysis of RNA ends (PARE), has been developed and used in plants for miRNA discovery and target validation (Addo-Quaye et al., 2008; German et al., 2008).

miRNAs may also initiate regulatory cascades that have a massive effect on the regulation of gene expression, reinforcing miRNA activity and ensuring gene expression homeostasis of large gene families. These cascades involve secondary siRNAs that associate with AGO proteins, similarly to miRNAs. Secondary siRNAs arise predominantly from RNAs that are targeted and cleaved by 22-nt- (Cuperus et al., 2010) or, if containing two target sites, 21-nt-miRNAs (Axtell et al., 2006). In the former case, the 22-nt miRNA duplex induces a conformational change in the AGO1 protein that allows the RNA-induced silencing complex (RISC) to recruit an RNA-dependent RNA polymerase (RDR6) and other components necessary for secondary siRNA production (Yoshikawa et al., 2005; Manavella et al., 2012). RDR6 converts the targeted RNA into long, double-stranded RNA which is then processed by Dicer-like protein 4 (DCL4) into secondary siRNAs mainly in a 21-nt-phased register starting from the miRNA cleavage site (Chen et al., 2007). The biogenesis of secondary siRNAs extends toward regions upstream or downstream of the initial target site, and the extension in the 5′- to 3′-direction is frequently observed (Aregger et al., 2012). Bioinformatics and molecular biology studies have shown that secondary siRNAs originate from non-coding, transacting siRNA loci such as those discovered at first in Arabidopsis (Peragine et al., 2004; Vazquez et al., 2004). In addition, secondary siRNAs also originate from coding genes of pentatricopeptide (Howell et al., 2007), auxin receptors (Si-Ammour et al., 2011; Windels and Vazquez, 2011), and NBS–LRR clades (Shivaprasad et al., 2012). These secondary siRNAs were shown to have roles in the expression of genes belonging to the same gene families since they can target paralogs in homologous regions.

Plant virus infections often result in onset of symptoms that are reconcilable with virus-induced alterations of RNA silencing-based endogenous pathways, due to: (i) the direct activity of viral silencing suppressors on endogenous sRNAs or on silencing related effectors (Csorba et al., 2015); (ii) the abundance of virus-deriving (v)-siRNAs in competition with endogenous sRNAs; (iii) the action of specific v-siRNAs entering into RNA silencing complexes and targeting specific host genes (Shimura and Pantaleo, 2011; Miozzi et al., 2013a); and (iv) potential functionalities of endogenous siRNAs triggered by viral infections (i.e., va-siRNAs, Cao et al., 2014). Tomato yellow leaf curl Sardinia virus (TYLCSV) is a *Begomoviru*s of the family *Geminiviridae*. Geminiviruses are circular single-stranded (ss)DNA viruses forming mini-chromosomes associated with cellular histones and are transcribed and replicated in the nucleus through dsDNA intermediates (Rojas et al., 2005; Brown et al., 2012). Geminiviruses encode viral suppressors of RNA silencing that are known to interfere either with DNA methylation (Buchmann et al., 2009; Yang et al., 2011; Zhang et al., 2011) or with the biogenesis of RDR6-dependent secondary siRNAs (Glick et al., 2008). Geminiviruses belonging to the *Begomovirus* genus have been extensively studied for their capacity to alter the accumulation of specific conserved miRNAs in infected tissues, resulting in the modification of the translation landscape of genes involved in the development of plants (Naqvi et al., 2010; Amin et al., 2011). TYLCSV induces specific systemic yellowing in tomato through a mechanism that has yet to be fully characterized.

Plant miRNA targets fall within gene families whose members have largely redundant functions and are mostly present in plant genomes in several copies. Whole genome duplication is a significant aspect in the evolution of eukaryotes, and evidence shows that most, if not all, angiosperms have undergone at least one ancient genome-doubling event in their evolutionary history (Vanneste et al., 2014). As such, many paralogs show divergence in gene structure, expression pattern, and function. Furthermore, two paralogs may diverge in their miRNA binding sites and surroundings, which may impact their fine expression and function (Wang and Adams, 2015). Recently, it has been shown that *Nicotiana benthamiana* possesses two functional *NbAGO1*-like paralogs. One 18-nt-long insertion/deletion (indel), which is located in the immediate vicinity of the miR168 target site, considerably affects the miR168-guided, post-transcriptional regulation of *NbAGO1* mRNAs. The indel effect is underpinned under conditions of viral infection. Indeed, *NbAGO1* homeologs are redundantly involved in their susceptibility to viral infection but are divergently involved in the phenotype associated with the infection (Gursinsky et al., 2015).

In this report, we diagnosed miRNA-mediated cleaved transcripts in both healthy and virus-infected plants through PARE. We integrated the experimental data with publicly available annotations and gained insight into the parallel evolution of gene families and their miRNA-mediated transcriptional regulation. We focused our attention on those targets of conserved miRNAs (i.e., those commonly shared among flowering plants) that were exclusively revealed in virus-infected plants and identified their closest evolutionary paralogs. Concerning the capacity to reveal the miR168-mediated cleavage of a longer *sly-AGO1* transcript (containing a 21-nt indel downstream the miRNA target site), we found that geminivirus infection in tomato parallels previous observations in tombusvirus-*N. benthamiana* (Gursinsky et al., 2015). Similarly, we revealed several cases of duplicated genes that are distinguished by 3-nt to 49-nt indels close to miRNA recognition sites and, in most cases, only paralogs lacking the indel were cleaved, being *bona fide* more vulnerable to miRNA-mediated cleavage. Within specific targets, our study revealed that one mRNA transcript coding for a CC–NBS–LRR protein holds both the miR482 and miR6024 target sites, which overlap to some extent. By mRNA-seq and PARE analysis we found that the transcript is significantly down-regulated and cleaved only in virus-infected plant tissues. Moreover, 21- to 22-nt-long secondary siRNAs were found associated with it either in a phased register or not. Unexpectedly, none of the secondary siRNAs from the miR6024-cleaved NBS–LRR were involved in cascade regulation of NBS–LRR paralogs; instead, some of them were involved in regulation of other disease proteins. Surprisingly, a group of these secondary siRNAs explained cleavages of several mRNAs coding for factors implicated in photosynthesis processes; this finding could explain the typical yellowing symptomatology induced by TYLCSV infection, thus suggesting a novel molecular mechanism involved in plant symptom development associated with biotic stresses.

## Materials and Methods

### Plant Material, Virus Inoculation, Nucleic Acid Extraction, RNA and DNA Gel Blots

Plants of *S. lycopersicum* L. cultivar “Moneymaker” were inoculated, as previously described, either by TYLCSV or by mock (Miozzi et al., 2013b). Plants were maintained in an insect-proof greenhouse at temperatures of 20–28°C/16–20°C (day/night), with a 16/8-h (light/dark) photoperiod and supplementary lighting. A minimum of six leaves per plant (from six plants, three biological replicates/thesis) were collected at the appearance of yellowing and curling symptoms typical of TYLCSV infection. Samples were ground in liquid nitrogen and used for total RNA extraction. RNAs were extracted using the Sigma RNA plant kit. DNA contaminants were removed using the Turbo DNA-free kit (Bio-Rad).

For Northern blot analysis, 10 μg of total RNA from either mock-inoculated or virus-infected leaf tissue were resolved on 12% PAGE gels. Once transferred to the positively charged nylon membrane Hybond N+ (Roche Diagnostics), miR171, and miR168 were detected as internal controls with oligoprobes, as previously described (Pantaleo et al., 2010b), whereas functional *Solyc05g008070*-derived siRNAs (**Figure 7C**) were detected using a mixture of P^32^-labeled DNA antisense oligoprobes. Radiolabeled signals were observed by Storm 860 Molecular Imager.

DNA either from TYLCSV-infected or from mock-inoculated tomato leaf tissues was extracted as previously described (Noris et al., 1996). Two micrograms of DNA were resolved on 1% Agarose, 0.5X Tris–borate–EDTA, and transferred to the positively charged nylon membrane Hybond N+. Viral replication intermediates were detected using a P^32^-labeled probe generated by random priming on a CP-amplicon, as previously reported (Accotto et al., 2000).

### mRNA-seq, sRNA, and RNA-Ends Dataset Preparation and Bioinformatics Analysis

Two mRNA libraries for each of the two theses, virus-infected and mock-inoculated plant tissues, were obtained with Illumina strand-specific mRNA protocol sRNAs and sequenced. Libraries of sRNAs were produced using a TruSeq Small RNA Sample Kit (Illumina) and sequenced with standard sequencing oligos on the Illumina HiSeq 2500 platform. Differential expression of selected transcripts was confirmed by qRT-PCR (see later “Materials and Methods”).

The poly-A fraction of total RNA extracted from leaf tissue was analyzed for the identification of target transcripts of conserved miRNAs. To generate miRNA-cleaved target libraries (RNA-end-dataset) from leaf tissue of either mock-inoculated or virus-infected, symptomatic tomato plants we applied the previously described high-throughput experimental approach that identifies mRNAs targeted by miRNAs, also known as “PARE” (Addo-Quaye et al., 2008; German et al., 2009). PARE libraries were sequenced using 5′-GAGATCTACACGTTCAGAGTTCTACAGTCCGA-3′ as oligo on the Illumina HiSeq 2500 platform.

A quality check of the transcriptome was performed with FASTX toolkit^1^. mRNAs were aligned to the selected paralogous genes using PatMaN aligner with 0 mismatch (Prufer et al., 2008). A raw count of mapped reads, uniquely aligned on each one of the two paralogous genes considered, was used as input for the differential expression (DESeq) analysis (Anders, 2010). sRNA-seq fastq datasets were converted to fasta format, adapters were removed and selected for molecules 16- to 26-nt in length using the FASTX toolkit. Genomic and viral-derived siRNAs were identified by aligning filtered sRNAs to the Tomato genome [retrieved at solgenomics.net, (Fernandez-Pozo et al., 2015)] and viral Ref_Seq database available at the NCBI. Bowtie software was used for alignment allowing 0 mismatches (Langmead et al., 2009). miRNAs were identified using miRProf (Stocks et al., 2012) with perfect matches to sequences from the miRNA repository [miRBASE release 21, (Kozomara and Griffiths-Jones, 2014)]. Alignment of viral siRNAs to the TYLCSV genome (Accession number NC_003828.1) was performed as above and SAM output files were used as input in Geneious^®^ software 8.1.7 (Biomatters Ltd.) in order to obtain a graphical representation of viral siRNAs along the viral genome.

RNA-end libraries underwent the same bioinformatics flowchart indicated above for sRNAs, with the only difference being the 20- to 21-nt filter size that was used before performing the alignment.

### *Sly-AGO1* Cleavage Identification by 5′RACE and PARE Analysis

5′-RACE was performed as previously described (Gursinsky et al., 2015), except for the following: the 5′ adapter oligo used was described by German et al. (German et al., 2009), whereas the reverse specific oligo used is 5′-WTGGGWTGCASCTGCTTCTGG-3′. Cleaved *sly-AGO1a* and *sly-AGO1b* were discriminated based on the indels and SNPs revealed by Sanger sequence downstream of the cleavage target site. PARE datasets were aligned with the NCBI seq IDs of *sly-AGO1*a and *sly-AGO1b* using Bowtie with 0 mismatches. The SAM file output was used in Geneious^®^ software 8.1.7 (Biomatters Ltd.) to obtain the graphical representation.

### Bioinformatics for Identification of miRNA Targets in PARE Libraries

cDNA targeted by sRNA were identified using PAREsnp software (Folkes et al., 2012). miRNAs selected from sRNA datasets by miRProf (see above) and PARE datasets from Tomato mock-inoculated or TYLCSV-inoculated plant tissues were used as input in both cases. PAREsnp was performed using cDNA library ITAG2.4^2^.

The selected targeted cDNAs from PAREsnp analysis were searched in the Solgenomics platform (Fernandez-Pozo et al., 2015) and the SGNs associated with each gene model were then filtered out considering those from PAREsnp analysis. Orphan SGNs, i.e., those targeted by sRNAs but not associated with any gene model in ITAG2.4, were subsequently searched for in the Solgenomics datasets and queried with BLAST (Altschul et al., 1997) against the tomato genome cDNA (ITAG release 2.40) for a better annotation.

### Evolutionary Analyses, Conserved Protein Domain Predictions, mRNA-Targeting, and RNA Secondary Structure *in silico* Prediction

The gene orthology and paralogy predictions were downloaded from Ensembl (Ensembl Plants release 34—December 2016) [in EnsemblCompara GeneTrees: Analysis of complete, duplication-aware phylogenetic trees in vertebrates (Vilella et al., 2009)]. Both Plant Compara and Pan-Taxonomic Compara phylogenies were used to retrieve orthologous genes.

Protein domain predictions were obtained through the NCBI CD-Search (Marchler-Bauer and Bryant, 2004) searching against the CDD v3.14 database, and schematic representations were drawn by the CLC biogenomics workbench (QIAGEN Bioinformatics).

psRNATarget (Dai and Zhao, 2011) was used under default parameters. The preloaded small RNA “user-submitted transcripts” was chosen. Both *cleavage* and *translation* types of regulation were studied.

Centroid plain RNA secondary structure drawings and positional entropy were obtained using RNAfold with default parameters on the Vienna RNA website (Mathews et al., 2004; Gruber et al., 2008).

### Identification of Functional Secondary siRNAs

Identification of siRNAs with a phased register with the miRNA cleavage site on selected genes was obtained using the ta-si prediction tool from UEA small RNA workbench (Stocks et al., 2012) using as threshold a *p*-value of 0.01. A subtraction step of common sRNAs of mock-inoculated from TYLCSV-inoculated plant tissue dataset was run on Galaxy website^3^. The TYLCSV-infected plant tissue dataset filtered from the previous step was used in a PaTmaN alignment with 0 mismatches using sequences selected from mRNAs as reference (Prufer et al., 2008). TYLCSV-only mapping 21- and 22-nt-long siRNAs, either in a phased register or not, were used for a PAREsnp analysis as described above for miRNA target identification.

### Reverse Transcription Quantitative Polymerase Chain Reaction Assays

After quantification with Picodrop, the quality of RNA was checked using the Experion automated electrophoresis station (Bio-Rad). Total RNA (500 ng) was retro-transcribed with the High-Capacity cDNA Reverse Transcription Kit (Applied Biosystems). All quantitative reverse-transcription PCR (qRT-PCR) assays were carried out using iTaq Universal SYBR Green Supermix (Bio-Rad, CA, United States) in an apparatus CFX Connect Real-Time PCR Detection System (Bio-Rad, CA, United States). The comparative threshold cycle method was employed to calculate relative expression levels using tomato UBC (ubiquitin-conjugating enzyme, SGN-U582847) as the reference gene (Miozzi et al., 2014). The list of primers used is shown in **Supplementary Table S6**. All qRT-PCR reactions were performed using three biological replicates and two technical replicates.

## Results

### Genome-Wide Identification of sRNAs and Cleaved mRNAs in Virus-Infected and Virus-Free *S. lycopersicum*

**FIGURE 1:**
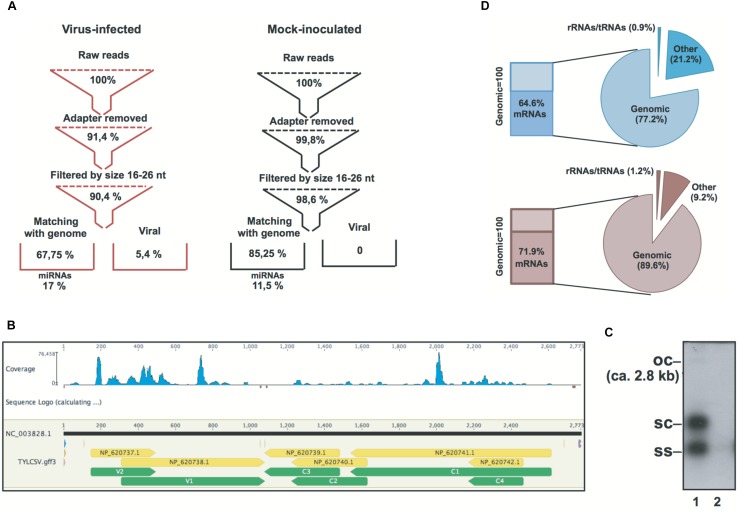
Statistics of *Solanum lycopersicum* genomic and non-genomic siRNAs in TYLCSV-infected and mock-inoculated plants. **(A)** Flowchart of sRNA datasets from raw data to genomic miRNAs and non-genomic viral sRNAs in virus-infected and mock-inoculated plants, respectively. **(B)** Distribution of TYLCSV-derived siRNAs in virus-infected plants.**(C)** Southern blot analysis of DNA from TYLCSV-infected tomato leaf tissue (lane 1) or from mock-inoculated tomato leaf tissue (lane 2). oc = open circle; sc = supercoiled; ss = single stranded. **(D)** Composition of PARE datasets in mock-inoculated and virus-infected plant tissues in terms of alignment or reads with rRNA, tRNAs, tomato genomic sequence, and tomato transcriptome (indicated in the figure as “mRNAs”).

Small (s)RNA datasets were obtained from the leaf tissue of mock-inoculated and TYLCSV-infected plants. Primary quality checks yielded a total of 17.8 million reads in the range of 18–34-nt (**Figure 1A** and **Supplementary Table S1**). sRNAs not matching the tomato genome were used in order to retrieve v-siRNAs, i.e., 0.7 million redundant reads in the case of TYLCSV-infected plants. Traces of v-siRNAs found in mock-inoculated plants, i.e., 71 in total, were not considered reliable marks of viral infection: they are rather siRNAs in datasets that likely align randomly with 0 mismatches with the all viral reference sequence (Ref_Seq) dataset. In this case, we almost found a 0 level of redundancy (69 unique out of 71 redundant, **Supplementary Table S1**), whereas v-siRNAs from full replicative viruses in infected tissues were characterized by a high level of redundancy due at least to the replication process and the production of secondary v-siRNAs (Pantaleo et al., 2010a; Miozzi et al., 2013b; Pirovano et al., 2014; Ghasemzadeh et al., 2018; **Figure 1B**). Southern blot hybridization on the total DNA extracted from plant tissues confirmed the infection and the active replication of the virus. Indeed, all TYLCSV replicative forms were detected only in virus-infected plant tissues (i.e., ssDNA, dsDNA, and open circle, **Figure 1C**, lane 1 vs. lane 2), indicating an active TYLCSV infection in the tissues analyzed.

PARE datasets were obtained from the same RNA preparations used for the sRNA libraries in line with previous reports (German et al., 2008; Arikit et al., 2014). RNA remnants of this size represent the 5′ ends of uncapped, poly-adenylated RNAs. Indeed, after initial processing, a small percentage of RNA remnants aligned to rRNAs/tRNAs (i.e., 0.9 and 1.2% for mock-inoculated and virus-infected plants, respectively) and the largest amount aligned to the *S. lycopersicum* genome (i.e., 77.2 and 89.6% for mock-inoculated and virus-infected plants, respectively). In addition, 64.6 and 71.9% of the reads aligning to the *S. lycopersicum* genomic sequence (for mock-inoculated and virus-infected plants, respectively) also aligned to the *S. lycopersicum* mRNA dataset (**Figure 1D**).

### Cleavage Remnants of Transcripts Coding for Transcriptional Factors and Disease-Resistance Proteins Are Found in Virus-Infected Plant Tissues

A total of 2.7 million redundant miRNAs were identified corresponding to only 250 and 251 unique miRNAs for TYLCSV-infected or mock-inoculated plants, respectively (**Figure 1A**). Some of the miRNAs analyzed were up-regulated in the presence of viral infection, while others were down-regulated in line with previous observations in Geminivirus-infected plants (**Supplementary Tables S1**–**S3**) (Naqvi et al., 2010; Amin et al., 2011).

The PARE datasets allowed us to assess the miRNA-mediated regulation of genes belonging to gene families coding for transcriptional factors and disease-resistance proteins, which are known to be altered under biotic stresses (Bartel, 2009). Therefore, we focused on transcriptional factors, such as Squamosa promoter binding proteins (SPL), Auxin response factors (ARF), Homeobox-leucine zipper proteins (HD-ZIPIII), nuclear transcription factors of the class of heme activator proteins (HAP), APETALA (AP) and related AP (RAP), and NBS–LRR-resistance factors. These gene families are regulated by miR156, miR160, miR166, miR169, miR172, and miR6024, respectively (Rhoades et al., 2002). Twenty-seven different genes from six different gene families were regulated by the above-mentionedsix miRNAs. Seventeen of them were cleaved in both mock-inoculated and virus-infected plants, while nine were targeted only in the virus-infected plants and only one was exclusively cleaved in the mock-inoculated plants (**Table 1**). We also interrogated psRNATarget (Dai and Zhao, 2011; Shao et al., 2014) and annotated the potential regulation of every single paralog belonging to each analyzed gene family. Twenty-five out of 27 genes from PARE analysis were common with psRNATarget (**Supplementary Tables S5**, **S6**).

**Table 1:** Target genes found to be cleaved by miRNAs in the PARE analysis either in mock-inoculated or virus-infected tomato plants.

miRNA name	Class of target genes	Gene	Symbol^a^	Found in virus-infected plants	Found in mock-inoculated plants
miR156	Squamosa	*Solyc02g077920*	Cnr	✓	✓
	promoter binding	*Solyc04g045560*	SlySBP2		✓
	proteins (SPL)	*Solyc05g012040*	SlySBP6b	✓	
		*Solyc05g015510*	SlySBP10	✓	✓
		*Solyc05g015840*	SlySBP13	✓	
		*Solyc10g009080*	SlySBP3	✓	✓
		*Solyc10g078700*	SlySBP15	✓	✓
miR160	Auxin response	*Solyc06g075150*	Sl-ARF10B	✓	✓
	factors (ARF)	*Solyc09g007810*	Sl-ARF16A	✓	
		*Solyc11g069500*	Sl-ARF10A	✓	✓
mirR166	Homebox-leucine	*Solyc02g024070*	–	✓	
	zipper proteins	*Solyc03g120910*	–	✓	✓
	(HD-ZIPIII)	*Solyc08g066500*	–	✓	✓
		*Solyc11g069470*	–	✓	
		*Solyc12g044410*	–	✓	✓
mirR168	Argonaute 1	*Solyc03g098280*	SlAGO1b	✓	
	(AGO1)	*Solyc06g072300*	SlAGO1a	✓	✓
miR169	Nuclear	*Solyc03g121940*	–	✓	
	transcription factors	*Solyc01g006930*	–	✓	✓
	(HAP)	*Solyc01g087240*	–	✓	✓
		*Solyc08g062210*	–	✓	✓
miR172	Transcription	*Solyc02g064960*	AP2b	✓	✓
	factors (AP and	*Solyc02g093150*	AP2c	✓	✓
	RAP)	*Solyc04g049800*	–	✓	✓
		*Solyc09g007260*	–	✓	
		*Solyc11g072600*	AP2d	✓	✓
miR6024	Disease resistance proteins (NBS-LRR)	*Solyc05g008070*	–	✓	

^a^*Data from Sol Genomics Network*.

Regarding NBS-LRRs targeted by miR6024, one gene belonging to this large family was specifically cleaved only in the virus-infected tissues, i.e., *Solyc05g008070* (**Table 1**). Worthy of note, *Solyc05g008070* has been previously described as targeted by miR482 (Shivaprasad et al., 2012) and, indeed, the psRNATarget reports four genes targeted by either miR6024 or miR482, (**Supplementary Table S3**), although PARE analysis never found 5′ remnants explained by miR482 for this gene family.

**FIGURE 2:**
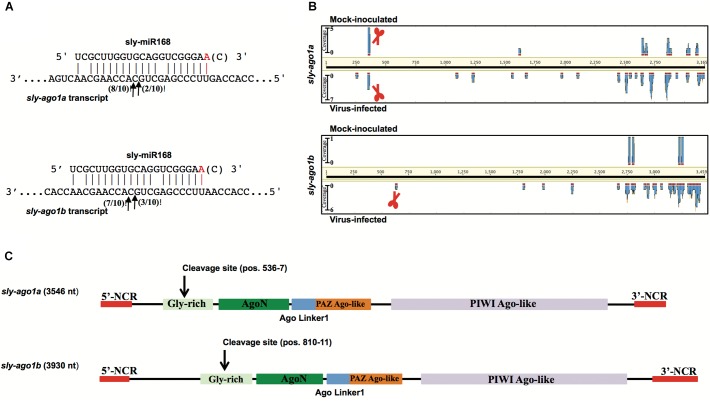
Degradation patterns of *sly-AGO1a* and *sly-AGO1*b. **(A)** 5′RACE of miR168-mediated cleavage of *sly-AGO1a* (NCBI seq ID NM_001279128.1) and *sly-AGO1*b (NCBI seqID NM_001279332.2) in TYLCSV-infected plants (upper panel) and *sly-AGO1a* in mock-inoculated plants. Arrows indicate 5′-remnants found in the analysis and with numbers are the clones sequenced. In red or in brackets sly-miR168 found in the datasets (**Supplementary Table S1**). **(B)** Degradation patterns of *sly-AGO1a* and *sly-AGO1*b (upper and bottom panels, respectively) in mock-inoculated and TYLCSV-infected tomato plants (upper and bottom profile in each panel, respectively). Schematic scissors indicate the miR168 cleavage sites. **(C)** Graph of *sly-AGO1a* and *sly-AGO1*b cDNAs showing the conserved protein domain and miR168 cleavage sites.

As a proof of concept, we extended the analysis also to mRNAs coding for AGO1. We performed a 5′-RACE analysis of miR168-mediated cleavage of transcripts from *sly-AGO1a* and *sly-AGO1b* and found that both transcripts can be cleaved at the same position (**Figure 2A**). Cleaved *sly-AGO1b* could only be found in the case of TYLCSV-infected plants (**Figure 2A**, lower panel and **Table 1**) and not in mock-inoculated plants. It is noteworthy that, the “A” at the 3′-end of sly-miR168a-5p v2 ensures pairing with the target site in both *sly-AGO1a* and *sly-AGO1b* transcripts (in red, **Figure 2A** and **Supplementary Table S2**).

The high-throughput analysis of *sly-AGO1a* and *sly-AGO1b* by PARE analysis confirmed what was found with 5′-RACE. **Figure 2B** shows the distributions of 5′-remnants found in mock-inoculated and in virus-infected plants along transcripts of either *sly-AGO1a* or *sly-AGO1b*. The degradation patterns of the two transcripts revealed a peak at the cleavage position, which was absent in the case of *sly-AGO1b* in mock-inoculated plants (**Figure 2B**, bottom vs. upper frame). The two *sly-AGO1* mRNAs differ in size: *sly-AGO1a* is 3,546 bp vs. 3,930 bp of *sly-AGO1b*; while the main difference is located at the 5′-terminal part of the coding sequence (**Figure 2C**).

### Indels Discriminate Paralogs Cleaved in Virus-Infected Plants

**FIGURE 3:**
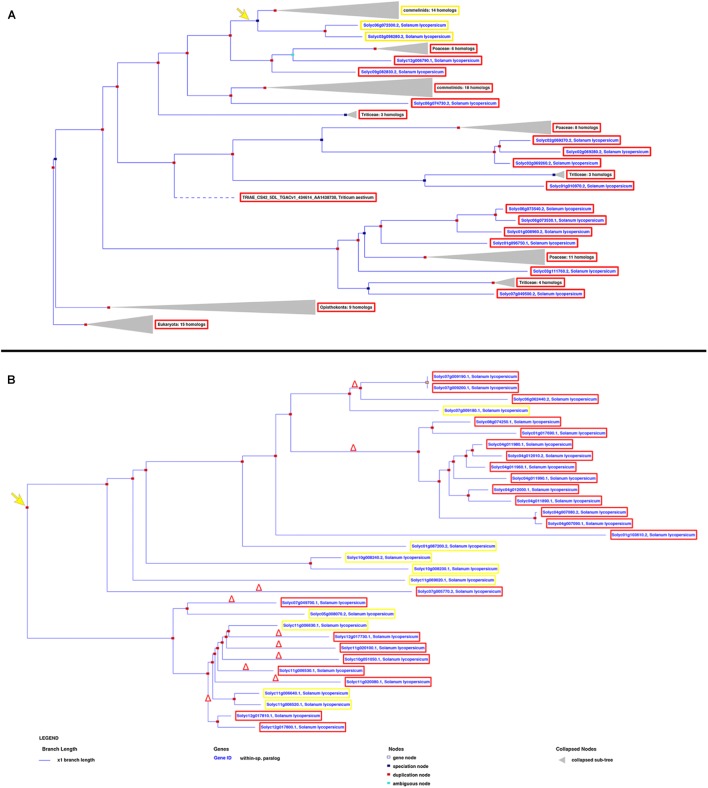
Phylogenetic trees showing the evolutionary history of AGOs **(A)** and NBS-LRRs **(B)** in *Solanum lycopersicum*. The yellow and red frames describe the genes harboring or not the miRNA recognition site, respectively, revealed by both PARE and *in silico* analyses. The yellow arrow indicates the evolutionary creation of the miRNA target site; the red delta symbol represents the deletion of the miRNA target site.

miRNA/target RNA recognition is a critical feature for miRNA functionality, target cleavage occurrence, and regulation of gene expression. *Sly-AGO1a* and *sly-AGO1b*, which differ by one 21-nt-long indel immediately downstream of the miR168 target site, have been previously highlighted in *S. lycopersicum* (Gursinsky et al., 2015) and here we confirmed that miRNA-mediated control is associated with the indel. To describe the diversity of miRNA targets among tomato genes that could explain the occurrence of cleavage events, we reconstructed each gene family based on its own evolutionary history, including orthologs, when available. Each gene tree is identified by an Ensembl Plant GeneTree ID; AGOs and NBS-LRRs are reported in **Figure 3**, whereas SPLs, ARFs, HD-ZIPIIIs, and AP/RAP are in **Supplementary Figure S1**. Upon examination of the phylogenetic trees, we retrieved the closest evolutionary paralogs to those targeted in PARE analysis only in virus-infected plant tissue (i.e., located after the duplication node, red squares in **Figure 3** and **Supplementary Figure S1**), and aligned their transcript sequences in pairs. This was carried out for all the gene families in **Table 1**, except for the HAP genes since the closest tomato paralog to HAP *Solyc03g121940* (cleaved in PARE analysis) is not annotated in the region of the miR169 recognition site (i.e., 3′UTR of *Solyc12g009050*, **Supplementary Figure S1A** and **Supplementary Table S3**).

**FIGURE 4:**
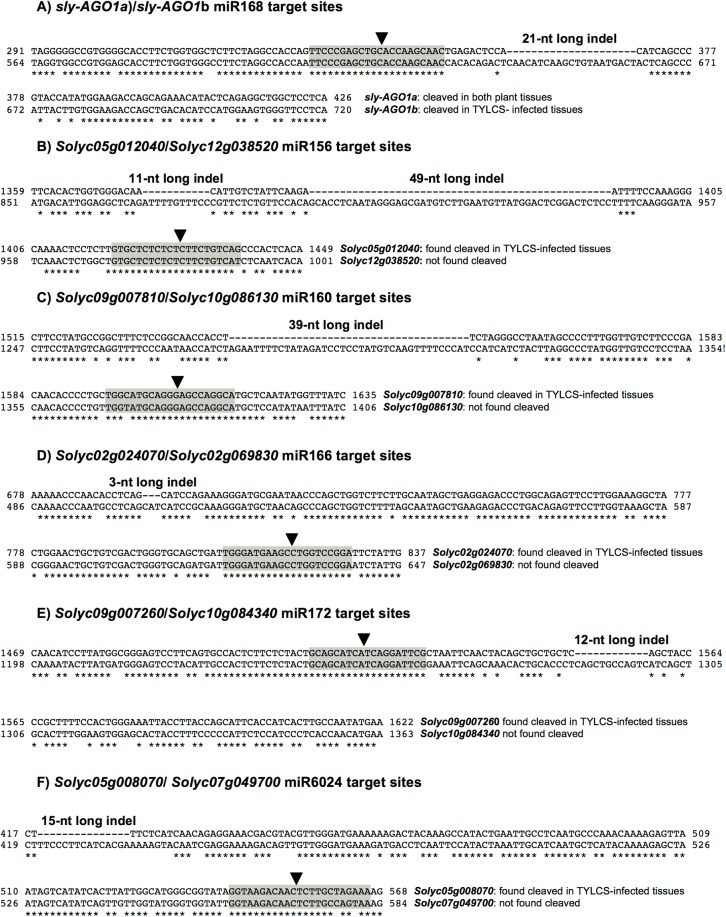
Alignments of miRNA target sites and indels. Alignments of close paralogs in the miRNA target site vicinity showing the presence of short indels. Black arrows indicate the miRNA cleavage site found in the PARE analysis. **(A)** sly-AGO1a/sly-AGO1b alignment with respect to miR168 target site; **(B)** Solyc05g012040/Solyc12g038520 alignment with respect to miR156 target site; **(C)** Solyc09g007810/Solyc10g086130 alignment with respect to miR160 target site; **(D)** Solyc02g024070/Solyc02g069830 alignment with respect to miR166 target site; **(E)** Solyc09g007260/Solyc10g084340 alignment with respect to miR172 target site; and **(F)** Solyc05g008070/Solyc07g049700 alignment with respect to miR6024 target site.

S*ly-AGO1a* and *sly-AGO1b* confirmed the presence of a 21-nt-long indel, 9-nt downstream of the miR168 target site (**Figure 4A**; Gursinsky et al., 2015). Surprisingly, also the pairwise alignment of all gene classes revealed the presence of indels close to the miRNA-target site. Indeed, we found (i) two consecutive indels of 11 and 49 nt in length, respectively, located 26 nt upstream the miR156 recognition site in the case of SPLs (**Figure 4B**); (ii) a 39-nt-long indel 48 nt upstream the miR160 cleavage site for ARFs (**Figure 4C**); (iii) a 3-nt-long indel, 113 nt upstream the miR166 recognition site in the case of HD-ZIPIIIs (**Figure 4D**); and (iv) a 12-nt-long indel, 24 nt downstream the miR172 recognition site in AP/RAP (**Figure 4E**).

Finally, alignment of *Solyc05g008070* (cleaved in PARE analysis in virus-infected plants, **Table 1**) to *Solyc07g049700* (its closest paralog in the NBS–LRR family, not cleaved in PARE analysis, **Supplementary Table S3** and **Supplementary Figure S1E**) disclosed a 15-nt-long indel, 123 nucleotides upstream of the miR6024 recognition site (**Figure 4F**).

**FIGURE 5:**
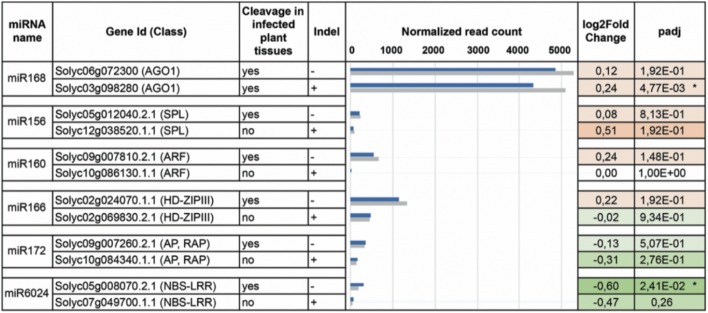
DEseq analysis. Paralogs for each miRNA-targeted (column 1) gene family (column 2) have been analyzed by DESeq. Corresponding transcripts are either cleaved in the virus-infested tissues or not (column 3). For each family, only one of the two paralogs contains the indel (column 4). In column 5 the normalized read counts for mRNAs purified from both virus-infected (gray bars) and mock-inoculated (blue bars) tissues. In column 6 and 7, log2FoldChange and adjusted *p*-values are displayed in the heatmap: red and green for up- and down-regulated transcripts. Asterisks indicate statistically significant variations that were also confirmed by qRT-PCR (three biological replicates and two technical replicates).

PARE analysis can verify target cleavage events, although it is not sufficient to exclude miRNA-mediated regulation of paralogs that were not found cleaved (**Figure 4**). We, therefore, set up transcriptome quantification via mRNA-seq, of selected target mRNAs, to confirm the presence/absence of miRNA targeting in the mock and infected plant tissues. All paralogs with no indels were significantly more expressed than those having indels in either mock or virus-infected tissues (**Figure 5**, blue and gray bars of histogram, respectively). When comparing the expression of each couple of paralogs in virus-infected vs. mock-inoculated plant tissues we found significant variations only in the case of *sly-AGO1b* and *Solyc05g008070*; the latter was down-regulated in virus-infected plant tissues (**Figure 5**, dark green with asterisk in the heatmap). Thus, we highlighted a possible correlation between the cleavage event (**Table 1**), the indel (**Figure 4**), and the down-regulation (**Figure 5**) in the case of the NBS–LRR transcript *Solyc05g008070*.

When predicting the secondary structure of the stretches of sequence in **Figure 4F**, which encompass both the 15nt indel and the miR6024 target site, we observed a sharp impact of the indel on the secondary structure and on the entropy at the target sites. The global-free energy of the indel-containing region was lower than in the case of the indel-lacking region (**Supplementary Figure S2**, panel B vs. A), and the artificial insertion of the indel in the latter strongly affected the structure (**Supplementary Figure S2C**). We also annotated the location of miRNA target sites in the gene structures with respect to the functional protein domains (**Supplementary Figure S2**, lower panels). Therefore, the functional indels described could impact secondary structures of the transcript rather than protein domains (see later in “Discussion”).

### The Indel-Containing miRNA-Targeted Paralogs in Virus-Infected Plants Generate Secondary siRNAs

pecific plant endogenous cascade networks can be triggered by miRNAs associated exclusively with the virus infection. We, therefore, identified all the 21–22-nt secondary siRNAs deriving from mRNAs targeted by miRNAs and solely present in virus-infected plant tissues. We first searched for those conserved miRNAs that were present in the 22-nt-long form in our siRNA libraries (**Supplementary Table S2**) that explained cleavage events in our PARE analysis (**Table 1**). We thus restricted the analysis to HD-ZIPIII, HAP, AP/RAP, and NBS–LRR transcripts cleaved by miR166, miR169, miR172, and miR6024, respectively. Given that AGO1 mRNA is also known in *Arabidopsis* and tomato to have secondary siRNAs initiated by miR168 (Mallory and Vaucheret, 2009; Shivaprasad et al., 2012), we focused on *sly-AGO1a* and *sly-AGO1b*. We included the tomato transcript *Solyc02g036270* coding for an NBS–LRR transcript targeted by miR482 as control for the effectiveness and reliability of the analysis. Indeed, *Solyc02g036270* was previously described as a producer of massive functional phased secondary siRNAs in tomato plants (Shivaprasad et al., 2012).

**FIGURE 6:**
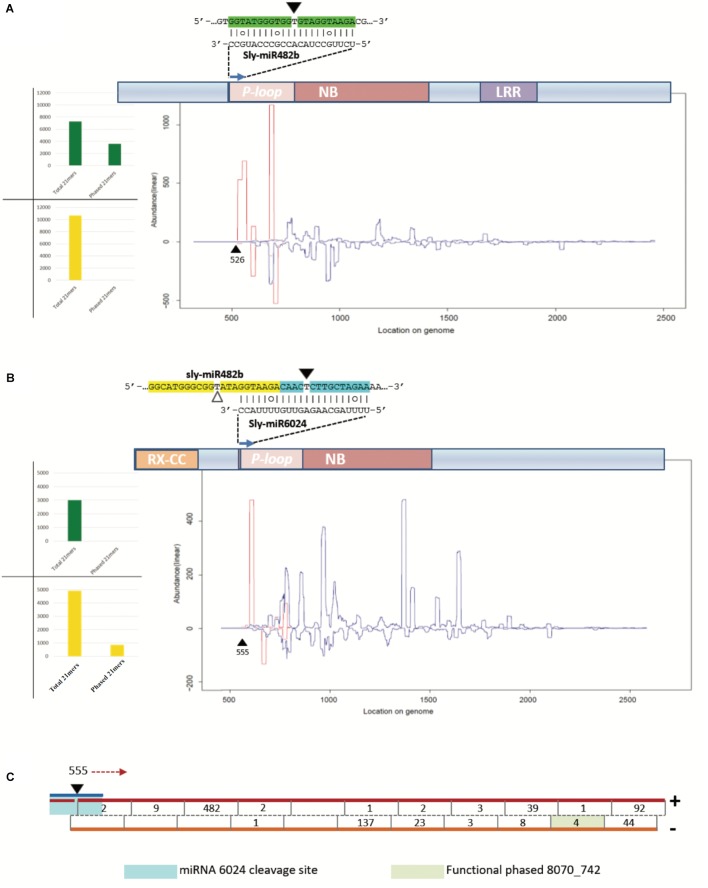
Secondary siRNAs initiated by miRNAs. **(A)** Secondary siRNAs initiated by miR482 on *Solyc02g036270* mRNA in mock-inoculated plant tissues; **(B)** secondary siRNAs initiated by miR6024 on *Solyc008070* mRNA in TYLCSV-infected plant tissues; **(C)** and portion of *Solyc05g008070* that is recognized as a source of phased secondary siRNAs. In **(A,B)** graphics of NBS–LRR genes are shown, which include conserved domains P-loop, nucleotide binding site (NBS), leucine-rich repeat (LRR), and RX-coiled-coil (RX-CC). In the graphic below the transcripts are indicated siRNAs of 21 nt in length, starting from the miRNA cleavage site (black triangle). White triangles indicate uncleaved miRNA target sites. Red and blue lines represent the abundance of each nucleotide position of phased and unphased 21mers, respectively. Histograms in **(A,B)** show total numbers of phased and unphased 21mers mapping to each gene locus in mock-inoculated (green) and virus-infected (yellow) plant tissues. In **(C)**, the blue line indicates miR6024, the black triangle the cleavage site in position 555, and red arrow indicates the direction of precursor processing into phased secondary siRNAs which are indicated by a black line in the double-stranded red/orange precursors. Numbers indicate abundance of each phased siRNA in the dataset.

We aligned the siRNA datasets uniquely from virus-infected plant tissues against selected mRNAs; the output is reported in **Table 2**. Massive amounts of secondary siRNAs derived from NBS–LRR genes and, as previously reported, a large number of siRNAs mapped to miR482-cleaved *Solyc02g036270* (Shivaprasad et al., 2012), i.e., 1,049 (corresponding to 724 unique reads). Similarly, *Solyc05g008070* targeted by miR6024 only in virus-infected tissues was covered by 936 siRNAs (corresponding to 665 unique reads). None or trifling reads mapped to *Solyc02g024070* and *Solyc11g069470* transcripts of the HD-ZIPIII clade, respectively. A modest number of siRNAs mapped to either *sly-AGO1a* or *sly-AGO1b* transcripts in agreement with a previous report (Mallory and Vaucheret, 2009). A similar situation was found for miR160-cleaved *Solyc09g007810*. In summary, we revealed secondary siRNAs associated mainly with those transcripts that were significantly affected in cleavage and expression in virus-infected plant tissues, i.e., *sly-AGO1b* and *Solyc05g008070* (see previous paragraph and **Figure 5**). Within the selected mRNAs in **Table 2**, we identified only two cases of phased register with respect to the miRNA target site: miR482/*Solyc02g036270* (**Figure 6A**) and miR6024/*Solyc05g008070* (**Figure 6B**). Surprisingly, phased siRNAs from *Solyc02g036270* were only present in mock-inoculated plant tissue datasets and not in the case of virus-infected tissues (histogram in **Figure 6A**). Phased siRNAs from *Solyc05g008070* were detected only in TYLCSV-infected plant tissues where miR6024 was active (histogram in **Figure 6B** and **Figure 6C**), despite the fact that the majority of secondary siRNAs from the two NBS–LRR transcripts were not in phase (**Figures 6A,B**, blue lines vs. red lines). Interestingly, we found that the miR6024 target site overlapped to some extent with that of miR482 (**Figure 6B**), which is not functional according to PARE analysis.

**Table 2:** Total and phased siRNAs associated with selected targets of miR160, miR168, miR169, miR482, and miR6024 and their functionality by PARE analysis in TYLCS-infected plants.

Gene (target of miRNA)	Class or Symbol	*n*° Associated siRNAs (Redundant/unique)	Functional by PARE analysis (Redundant/unique)		
			21nt unphased	21nt phased^a^	22nt
*Solyc09g007810* (miR160)	ARF transcription factor	46/40	–	–	–
*Solyc02g024070* (miR166)	HD-ZIP transcription factor	0	–	–	–
*Solyc11g069470* (miR166)	HD-ZIP transcription factor	4/4	–	–	1
*Solyc03g098280* (miR168)	*sly-AGO1b*	30/26	–	–	1
*Solyc06g072300* (miR168)	*sly-AGO1a*	0	–	–	–
*Solyc02g036270* (miR482)	NBS-LRR	1049/724	31/22	–	22/15
*Solyc05g008070* (miR6024)	NBR-LRR	936/665	21/17	4/1	26/13

^a^*Register starting from miRNA cleavage*.

### A Discrete Number of Secondary siRNAs From the NBS–LRR Family Are Functional in Trans

Secondary siRNAs including those in a phased register are known to be functional upon incorporation into RISC complex (Vazquez and Hohn, 2013). Being PARE datasets a global analysis of the cleaved transcripts, we performed a dedicated analysis searching 5′-remnants of mRNAs explained by siRNAs in **Table 2**. Functional siRNAs of 21 and 22 nt are detailed in **Supplementary Tables S4**, **S5**, respectively, and are summarized in **Table 2**. Among functional secondary siRNAs in TYLCS-infected plant tissues deriving from *Solyc02g036270*, we could find 22 and 15 unique functional siRNAs of 21 and 22 nt in length, respectively. Instead, one phased, seventeen 21-nt-long un-phased and thirteen 22-nt-long non-redundant functional siRNAs were associated with *Solyc05g008070* (**Table 2** and **Supplementary Tables S4**,**S5**).

The 21-nt-long siRNA indicated as siRNA 8070_742 in **Figure 6C** (pale green box) derived from a highly statistically significant (*P*-value = 1,068e^-8^) phased locus starting at position 555 of *Solyc05g008070*, which corresponds to the miR6024 cleavage site (**Figures 6B,C**). siRNA 8070_742 explained the cleavage of *Solyc03g083250* coding for a putative ATP-dependent CIp protease.

At least one secondary siRNA deriving from NBS–LRR genes has been shown to target other mRNAs of defense-related proteins (Shivaprasad et al., 2012); this was confirmed in our analysis, but we could not find any NBS–LRR transcript among the targets. Indeed, we identified targeted host factors previously described in literature as involved in plant pathogen-related molecular patterns, such as glucan-endo-1-3-beta-glucosidase (Bucher et al., 2001), polyphenol oxidase (Kikuchi and Yamaguchi, 1960), and aquaporin-like proteins (Sade et al., 2014; **Supplementary Tables S4**, **S5**).

**FIGURE 7:**
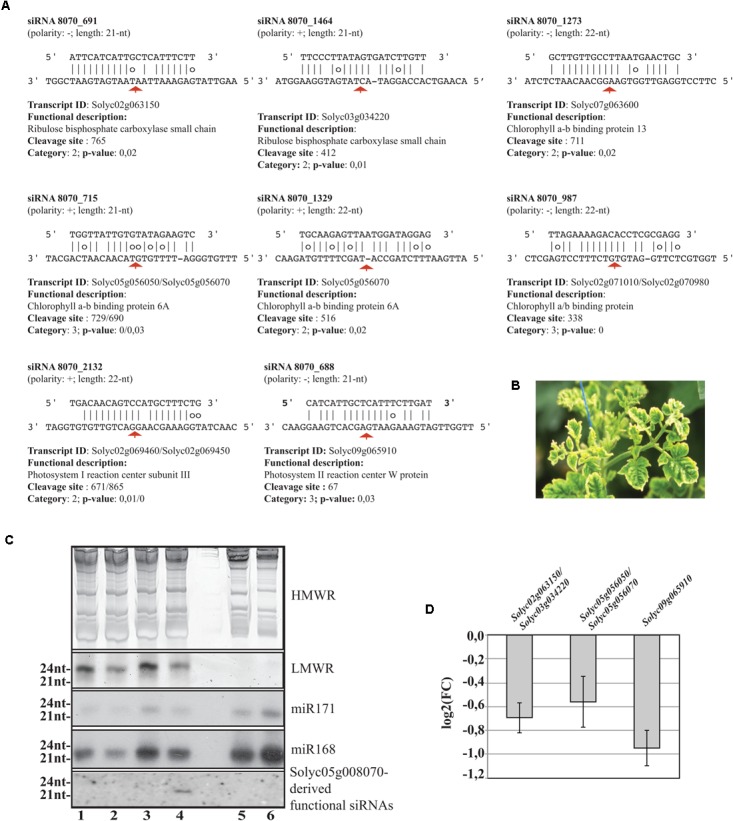
Functional secondary siRNAs and expression of genes involved in photosynthesis. **(A)** Functional 21nt and 22nt-long secondary siRNAs from the NBS–LRR transcript *Solyc05g00807*0 and targeted transcripts. Transcripts sharing conserved miRNA target sites are indicated and separated by “/”. Red arrows indicate the cleavage sites. **(B)** Typical leaf yellowing induced by TYLCSV in virus-infected tissues. **(C)** Short RNA Northern blot analysis. High- or low-molecular weight RNAs patterns (HMWR and LMWR, respectively). Lanes 1 and 3: mock-inoculated *Solanum lycoperscum*, Lane 2: total RNA from TYLCSV-inoculated plant tissues not showing yellowing sympthoms, Lane 4: total RNA from TYLCSV-inoculated plant tissues showing typical yellowing symptoms. Lanes 5 and 6, *Arabidopsis thaliana* and *Nicotiana benthamiana* total RNA, respectively. **(D)** qRT-PCR (three biological replicates and two technical replicates) for the expression of *Solyc02g063150*/*Solyc03g034220* (1), *Solyc05g056050*/*Solyc05g056070* (2), and *Solyc09g065910* (3) transcripts in TYLCSV-infected tissues from tomato plants. Expression values (bars ± SE) for each transcript or paralogs are given as log2 of fold change (FC) relative to control tissues from mock-inoculated plants.

Worthy of note, 11 out of 42 targeted mRNAs were key players of plant photosynthetic machinery. In **Figure 7A**, we report eight secondary siRNAs from *Solyc05g008070* originated downstream of the miR6024 cleavage site uniquely present in virus-infected plant tissues that target and cleave core members of photosystem I and II, chlorophyll-binding proteins, and ribulose bisphosphate carboxylases (RuBiSCO). Conventional and specific Sanger sequenced 5′-RACE were also performed on the library besides the high-throughput sequencing. Validation by Northern blot analysis revealed the expression of the secondary functional siRNAs only in the case of RNAs from TYLCSV-inoculated plant tissues showing typical yellowing symptoms (**Figures 7B,C**, lane 4). No secondary functional siRNAs were detected in either mock-inoculated (**Figure 7C**, lanes 1 and 3) or TYLCSV-infected plant tissues not-showing yellowing symptoms (**Figure 7C**, lane 2). miR168 and miR171, used as a control of the Northern analysis, were detected in all the samples under investigation (**Figure 7C**). Finally, DESeq analysis resulted in down-regulation of all targeted transcripts in TYLCSV-infected plant tissues. For those transcripts that could be investigated by qRT-PCR, a significant 1.5- to 2-fold down-regulation in virus-infected vs. mock-inoculated plant tissues, thus confirming the functionality of these siRNA cleaving targets solely associated with virus infection (**Figure 7D**).

## Discussion

Plant viruses are infectious entities that alter gene expression and are therefore recognized as “phenotype extenders.” For instance, it has been shown that virus infection can enhance plants’ attractiveness to pollinators (Groen et al., 2016) as well as the resilience of susceptible plants to drought (Xu et al., 2008; Westwood et al., 2013; Pantaleo et al., 2016) and that they defend plants from several species of herbivores (Gibbs, 1980; Pan et al., 2013). The fundamental reasons for this gene regulation are not yet fully understood, but many phenomena can be ascribed to a mechanistic role for miRNAs in regulating innate immunity in the case of plant–pathogen interaction (Katiyar-Agarwal and Jin, 2007; Deng et al., 2018). Moreover, plant endogenous siRNAs associated with viral infection denoted as va-siRNAs are surely implicated in the regulation of genes in plants under virus attack (Cao et al., 2014).

In plants, fungi, and animals, indel studies have led to understand how proteins function as well as to the discovery of useful experimental and drug targets. Moreover, indels are recognized as having significant value as phylogenetic markers. For selected miRNA targets, we show that short indels upstream or downstream in the vicinity of miRNA targets can discriminate between the miRNA cleavage events of close paralogs. Indeed, *S. lycopersicum* possesses two *AGO1* homologs named *sly-AGO1a* and *sly-AGO1b*, differing for 21-nt-long indels just downstream of the miR168 target site. *Sly-AGO1b* (the transcript containing the insertion) is cleaved in TYLCSV-infected plants, whereas it is not in mock-inoculated plants. Consistent data came from mRNA-seq analysis since the *sly-AGO1b* appeared significantly up-regulated in virus-infected tissues, which is in agreement with our previous observations (Gursinsky et al., 2015). This provides further support to specific roles ascribed to the two AGO1 paralogs even in a different plant-virus system. Herein, we reveal that several nuclear transcription factors are also cleaved by specific miRNAs only in the presence of the viral infection. Among these transcriptional factors, there are two SPL genes cleaved by miR156 (i.e., *sly-SPB6b* and *sly-SPB13*), one ARF gene cleaved by miR160 (*Sl-ARF16A*), two HD-ZIPIII genes cleaved by miR166 (*Solyc02g024070* and *Solyc11g069470*), one AP transcription factor cleaved by miR172 (*Solyc09g007260*), and one NBS–LRR disease-resistance protein-coding gene cleaved by miR6024 (*Solyc05g008070*). We show that 12-, 15-, 3-, and 39-nt-long single indels are in close vicinity to the target sites of miR172, miR6024, miR166, and miR160, respectively. Nevertheless, in all these cases, the indels discriminate between cleavages of close paralogs: the version containing the insertion was never cleaved, whereas the other was found cleaved in TYLCSV-infected plant tissues. All paralogs with no indels were significantly more expressed than those having indels in either virus-infected or mock plant tissue. A significant differential expression of paralogs in mock-inoculated tissues suggests fine and independent gene regulation that renders unclear the interpretation of the role of indels in miRNA-dependent regulation. However, *Solyc05g008070* lacking one 15-nt-long indel, 123 nucleotides upstream of the miR6024 recognition site, is cleaved and is down-regulated solely in virus-infected plant tissues. All the analyzed genes are homologs, thus they derive from a whole genome duplication and belong to different subgenomes (Tomato Genome, 2012); moreover, they are likely to have a redundant role. Hence, we hypothesized the possibility to regulate them differently, mediated by a distinctive sensitivity to the miRNA-mediated cleavage, might be skilfully exploited by the plant to convert an “excess of function” in a stress-resistance tool. Short indels are unlikely to have a significant impact on protein size and we show that they do not impair the recognition of functional domains. Instead, they are confirmed to play a role in the secondary structure of RNA influencing the access of RISC to target sites in line with previous *in vitro* and *in vivo* observations either in animal or plant systems (Ameres et al., 2007; Long et al., 2007; Schuck et al., 2013; Gursinsky et al., 2015). In the case of the indel-lacking paralog, *Solyc05g008070*, this is further corroborated by the *in silico* prediction of RNA folding and entropy at target site level.

The CC–NBS–LRR class of genes confers resistance to several plant viruses in an effector-specific interaction (Kachroo et al., 2006). miR482 targets tomato mRNAs for CC–NBS–LRR (i.e., *Solyc02g036270*) and the mRNA decay causes the production of secondary siRNAs that in turn down-regulate other mRNAs of disease-resistance genes: this has been proposed as a novel pathogen-inducible layer of plant defense (Shivaprasad et al., 2012). Here, we analyzed the production of secondary siRNAs from *Solyc02g036270* and we confirm that it produces phased register secondary siRNAs with respect to the miRNA target site, likely induced by the 22-nt-long miR482 found in our dataset. Importantly, phased siRNAs from *Solyc02g036270* were only present in mock-inoculated and not in virus-infected plant tissue datasets. The production of secondary siRNAs from *Solyc02g036270* requires RDR6 that is engaged in converting targeted RNA in dsRNA (Shivaprasad et al., 2012). In this pathway, suppressor of gene silencing 3 (SGS3) is also involved (Mourrain et al., 2000). Tomato yellow leaf curl virus (TYLCV), a close relative of TYLCSV, is known to produce the V2 protein that is recognized as a viral suppressor of RNA silencing (Zrachya et al., 2007); V2 directly binds SGS3 in plants and this interaction is required for V2 RNA silencing suppression activity (Glick et al., 2008). V2 is produced by the virus to counteract plant antiviral silencing but to date, no specific action has been described. Glick and colleagues (Glick et al., 2008) advanced the hypothesis that V2-mediated inactivation of SGS3 in infected tissues could explain “leaf curling” symptoms. Here, we reveal for the first time that the miRNA-mediated cleavage of the NBS–LRR transcript is not relieved in TYLCSV-infected plant tissues; instead, the production of secondary siRNAs in phased register is abolished, likely through the inactivation of SGS3/RDR6 by V2. The availability of genome-wide 5′-RACE analysis allowed us to show that in the absence of phased secondary siRNAs in TYLCSV-infected tissues no transcript of the NBS–LRR clade was found cleaved except by miRNAs. The loss of biogenesis of phased secondary siRNAs would reinforce the defensive effect and, noteworthily, our data suggest that such inactivation could happen downstream the miRNA-mediated cleavage hampering dsRNA synthesis by geminivirus viral suppression. Mechanisms of production of functional unphased siRNAs are at the moment unknown and deserve further investigations.

The data from the present study suggest for the first time that CC–NBS–LRR class of genes includes many resistance (R) genes could be directly implicated in symptom development through secondary cascade pathways. Indeed, one NBS–LRR transcript releases secondary siRNAs that target transcripts coding for RuBISCO, chlorophyll a/b binding protein 6A, and photosystem II reaction center W protein. These are significantly down-expressed in infected tissues at least 1.5-fold. In addition, Northern blot analysis unveiled the expression of these secondary sRNAs exclusively in plant virus-infected tissues showing yellowing. Thus, here we reveal a novel mechanism that could be added to earlier findings in virus–host interaction. Leaf chlorosis is often seen in plants infected with viruses and viroids and is known to be associated with a reduced content of chlorophyll in leaf cells (Dawson, 1992). As chlorophyll is responsible for the green color of leaves, chlorotic leaves range from pale to yellow, through yellow–white. Cucumber mosaic virus (CMV) yellowing (Y) satellite RNA (Y-sat) is a non-coding subviral RNA and modifies the typical symptom induced by CMV in specific hosts; direct evidence identified one 22-nt-long siRNA derived from Y-sat that modulates the yellowing symptom by RNA silencing-based regulation of tobacco magnesium protoporphyrin chelatase subunit I (ChlI), a key gene involved in chlorophyll synthesis (Shimura et al., 2011). In the case of grapevine infected by Grapevine fleck virus, v-siRNAs were able to target and cleave transcripts of the photosynthetic system Y (Miozzi et al., 2013a). However, the v-siRNAs-dependent pathogenesis mechanism is not general. Indeed, alteration of chloroplast development induced by Peach latent mosaic viroid (PLMVd) leads to albino phenotype (an extreme chlorosis symptom) (Rodio et al., 2007). In the case of PLMVd, the block of chloroplast development is likely due to the RNA silencing of one chloroplastic heat shock protein 90 (Navarro et al., 2012). Chlorosis is also due to the localization in chloroplasts of either viral proteins, such as in the case of Tobacco mosaic virus coat protein and Rice stripe virus disease-specific protein that hamper photosynthetic system II (Reinero and Beachy, 1989; Kong et al., 2014), or subviral RNAs such as in the case of Geminivirus betasatellites that severely alter chloroplast ultrastructure and induce vein clearing (Bhattacharyya et al., 2015).

The understanding of miRNA biology is still limited by our poor appreciation of their cell-type specific and spatio-temporal regulation. Many miRNAs exhibit discrete expression patterns and their regulation depends largely on the availability of particular AGO proteins and distinct target mRNAs within a given cell/tissue type. Next-generation sequence approaches to facilitate the simultaneous analysis of large gene sets but they typically provide a very limited spatio-temporal resolution of gene expression changes. Either in plants or animals (Kozomara et al., 2014), miRNA expression level is an unreliable indicator of miRNA repressive activity. In plants, laser microdissection coupled to RT-PCR showed that, on the contrary, the global pattern of mature conserved accumulation entails exquisite spatio-temporal variations in the transcription of nine distinct miRNA paralogs (Nogueira et al., 2007). The combinatorial analysis of small RNA sequencing and accurate expression data for target mRNA/proteins by the novel single-cell RNA-Seq analysis would solve either some discrepancies (such as miR6024 ∼2/3X less abundance in infected tissues than mock-inoculated tissues), or highlight the impact of secondary siRNAs controlling the expression of analyzed transcripts.

## Accession Numbers

The sRNA and PARE datasets have been deposited in GEO Omnibus under the entry code GSE93648. The DESeq analysis of selected paralogs was carried out using alignments deposited in SRA under the entry code SRP133920.

(To review GEO accession GSE93648:

Go to https://www.ncbi.nlm.nih.gov/geo/query/acc.cgi?acc=GSE93648

Enter token onsrwgugbxmpnwd into the box)

(To Access to metadata about SRA submission https://trace.ncbi.nlm.nih.gov/Traces/sra/?study=SRP133920).

## Author Contributions

VP designed the experiments and prepared PARE and sRNA datasets. LM prepared total RNA from tomato plants and performed quantification analysis. MC, CC, WP, and VP analyzed the data. MC, CC, and VP prepared the manuscript. All authors discussed the results and contributed to editing the paper.

## Conflict of Interest Statement

The authors declare that the research was conducted in the absence of any commercial or financial relationships that could be construed as a potential conflict of interest.

## Footnotes

1 http://hannonlab.cshl.edu/fastx_toolkit/index.html

2 ftp://ftp.solgenomics.net/tomato_genome/annotation/ITAG2.4_release/ITAG2.4_cdna.fasta

3 https://usegalaxy.org

## Supplementary Material

FIGURE S1 Phylogenetic trees showing the evolutionary history of selected gene families targeted by conserved miRNAs in *Solanum lycopersicum.* **(A)** *EPlGT00140000000465* evolutionary history for HAP genes; **(B)** *EPlGT00850000106226* evolutionary history for ARF genes; **(C)** *EPlGT00140000000795* evolutionary history for HD ZIPIII genes; **(D)** *EPlGT00850000106232* evolutionary history for AP/RAP genes; and **(E)** *EPlGT00820000103254* evolutionary history for SPL genes.

FIGURE S2 Impact of the functional indels on the secondary structures of the transcript. **(A,B)** Global-free energy of the indel-containing region (positions 360–622, *Solyc05g008070*) and of the indel-lacking region (positions 360–638, *Solyc07g049700*), respectively. **(C)** Impact of the artificial insertion of the indel in the indel-lacking region.

TABLE S1 Statistics of *Solanum lycopersicum* genomic and non-genomic siRNAs in tomato yellow leaf curl sardinia virus-infected and mock-inoculated plant tissues.

TABLE S2 Diversity of selected functional conserved miRNAs (expression profile in sRNA datasets from mock-inoculated and TYLCSV-infected tomato plants). Bold characters indicate nucleotides differing from the canonical miRNA sequence.

TABLE S3 *In silico* analysis of miRNA targets among the selected gene families, validated by PARE analysis.

TABLE S4 Output of PAREsnp listing 5′ RNA remnants of cDNA targeted by 21-nt-long secondary siRNAs in tomato TYLCSV-infected plant tissues.

TABLE S5 Output of PAREsnp listing 5′ RNA remnants of cDNA targeted by 22-nt-long secondary siRNAs in tomato TYLCSV-infected plant tissues.

TABLE S6 Oligonucleotides used for quantitative reverse transcription PCR experiments of selected transcripts in TYLCSV-infected vs. mock-inoculated tissues.
