# Supplementary figures and images for: A Short Indel-Lacking-Resistance Gene Triggers Silencing of the Photosynthetic Machinery Components Through TYLCSV-Associated Endogenous siRNAs in Tomato
Source: Front Plant Sci. 2018 Oct 11;9:1470. doi: 10.3389/fpls.2018.01470 (PMC6193080; doi:10.3389/fpls.2018.01470)

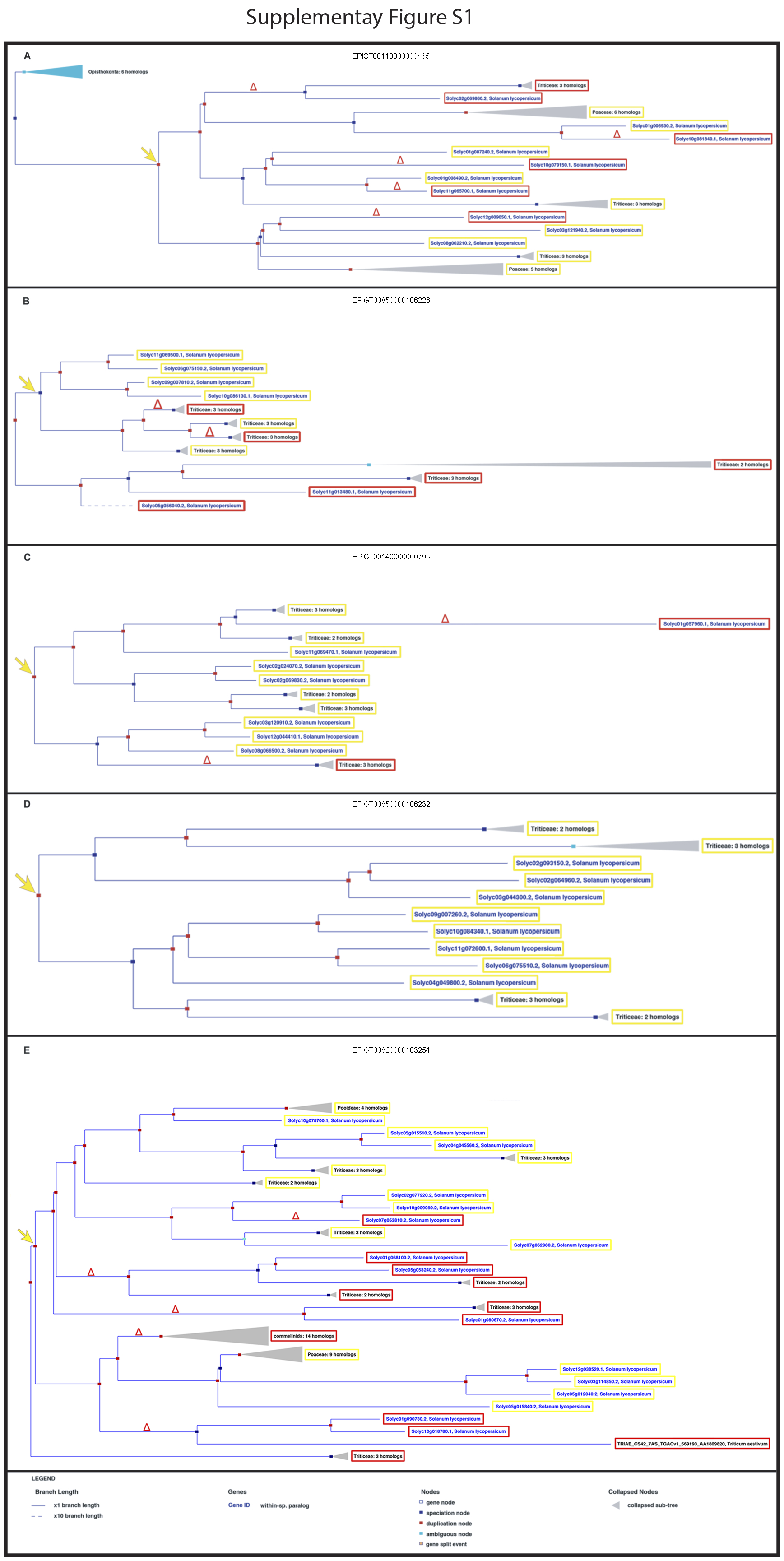

Supplement: FIGURE S1 — Phylogenetic trees showing the evolutionary history of selected gene families targeted by conserved miRNAs in Solanum lycopersicum. (A) EPlGT00140000000465 evolutionary history for HAP genes; (B) EPlGT00850000106226 evolutionary history for ARF genes; (C) EPlGT00140000000795 evolutionary history for HD ZIPIII genes; (D) EPlGT00850000106232 evolutionary history for AP/RAP genes; and (E) EPlGT00820000103254 evolutionary history for SPL genes. [file Image_1.TIFF]

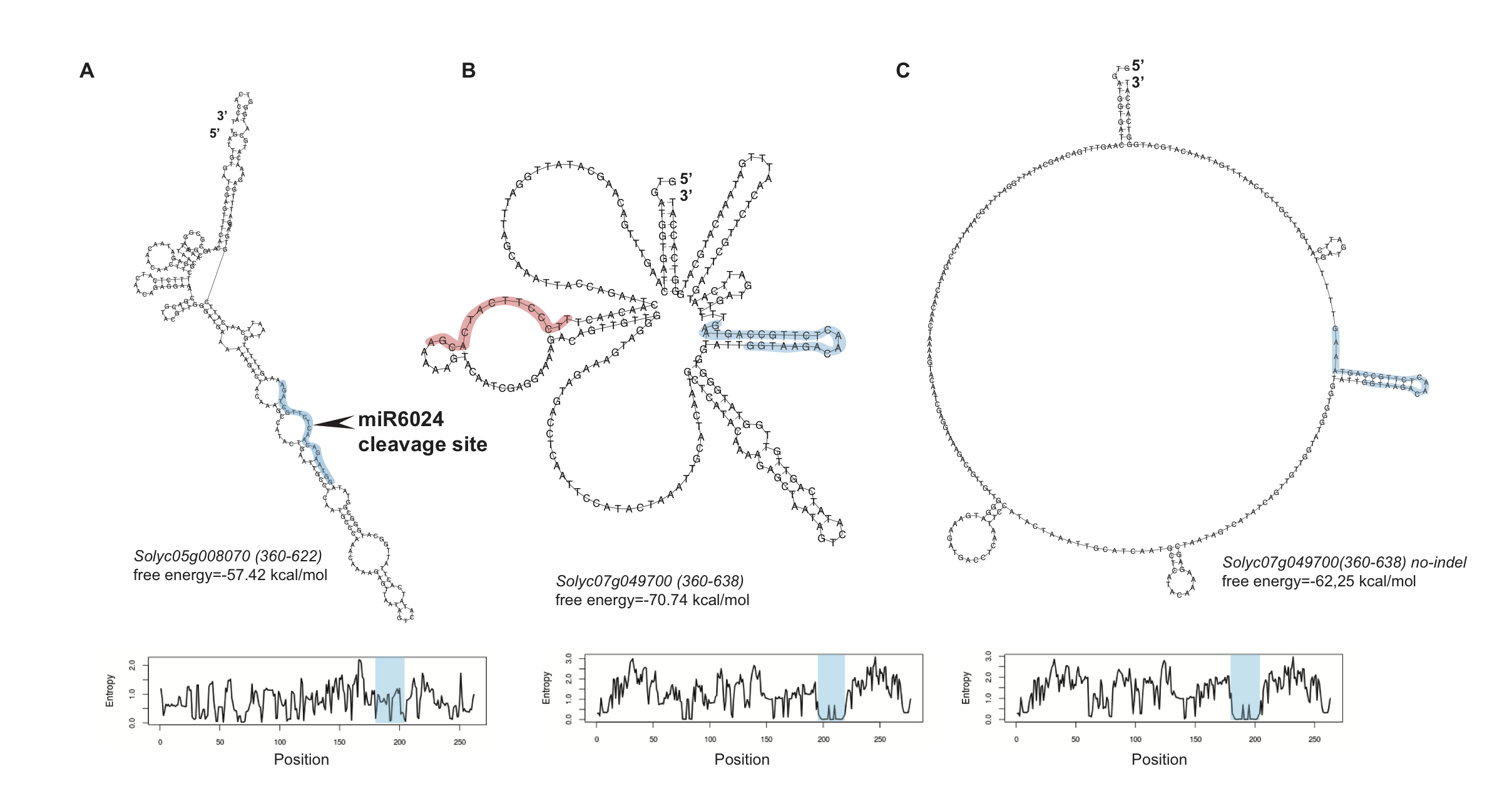

Supplement: FIGURE S2 — Impact of the functional indels on the secondary structures of the transcript. (A,B) Global-free energy of the indel-containing region (positions 360–622, Solyc05g008070) and of the indel-lacking region (positions 360–638, Solyc07g049700), respectively. (C) Impact of the artificial insertion of the indel in the indel-lacking region. [file Image_2.TIF]
