# Supplementary material for: A Short Indel-Lacking-Resistance Gene Triggers Silencing of the Photosynthetic Machinery Components Through TYLCSV-Associated Endogenous siRNAs in Tomato
Source: Front Plant Sci. 2018 Oct 11;9:1470. doi: 10.3389/fpls.2018.01470 (PMC6193080; doi:10.3389/fpls.2018.01470)
Supplement: TABLE S1 — Statistics of Solanum lycopersicum genomic and non-genomic siRNAs in tomato yellow leaf curl sardinia virus-infected and mock-inoculated plant tissues. [file Table_1.PDF]

**Supplementary Table S1.** Statistics of *Solanum lycopersicum* genomic and non-genomic siRNAs in Tomato yellow leaf curl sardinia virus-infected and mock-inoculated plant tissues.

| | | Redundant | | | Unique | | |
|---|---|---|---|---|---|---|---|
| | | **# of Reads** | **miRNAs or v-siRNAs** | | **# of Reads** | **miRNAs or v-siRNAs** | |
| | | | # of reads | % | | # of reads | % |
| **Virus-infected** | **Raw reads** | 13231170 | | | | | |
| | **Adaptor removed** | 12103878 | | | | | |
| | **Filter by sequence properties** | 11961919 | | | 2971654 | | |
| | **rRNA/tRNAexact matches removed** | 1356860 | | | 46244 | | |
| | **Matching with genome** | 8965078 | 2250590 | 25,10 | 2348893 | 251 | 0,01 |
| | **Non-Matching with genome** | 1639981 | 710341 | 43.31 | 18863 | 18863 | 0,37 |
| **Mock-inoculated** | **Raw reads** | 4479742 | | | | | |
| | **Adaptor removed** | 4472405 | | | | | |
| | **Filter by sequence properties** | 4428916 | | | 1643488 | | |
| | **rRNA/tRNAexact matches removed** | 220998 | | | 28454 | | |
| | **Matching with genome** | 3819266 | 516769 | 13,53 | 1349958 | 250 | 0,02 |
| | **Non-Matching with genome** | 388652 | 71 | 0,02 | 265076 | 69 | 0,03 |
