# Supplementary material for: A Short Indel-Lacking-Resistance Gene Triggers Silencing of the Photosynthetic Machinery Components Through TYLCSV-Associated Endogenous siRNAs in Tomato
Source: Front Plant Sci. 2018 Oct 11;9:1470. doi: 10.3389/fpls.2018.01470 (PMC6193080; doi:10.3389/fpls.2018.01470)
Supplement: TABLE S2 — Diversity of selected functional conserved miRNAs (expression profile in sRNA datasets from mock-inoculated and TYLCSV-infected tomato plants). Bold characters indicate nucleotides differing from the canonical miRNA sequence. [file Table_2.PDF]

Supplementary Table S2. Diversity of selected functional conserved miRNAs (expression profile in sRNA datasets from mock-inoculated and TYLCSV-infected tomato plants). Bold characters indicate nucleotides differing from the canonical miRNA sequence.

miRNA family	Similarity in miRBase*	Sequence (5'-3') (mature, star strands and variants)	Size	Mock-inoculated (ppm)	TYLCS-infected (ppm)
miR156	sly-miR156a,b,c	UUGACAGAAGAUAGAGAGCAC	21	847,04	609,22
	sly-miR156d-3p	GCUCUCUAUGCUUCUGUCAUC	21	121,69	65,19
		GCUCUCUAUGCUUCUGUCAUC**A**	22	-	0,55
	sly-miR156d-5p	UUGACAGAAGAGAGUGAGCAC	21	-	0,18
miR160	sly-miR160a	UGCCUGGCUCCCUGUAUGCCA	21	7,19	5,13
miR166	sly-mir166a	UCGGACCAGGCUUCAUUCCCC	21	168357,36	329312,16
		UCGGACCAGGCUUCAUUCCCC**C**	22	14,39	36,62
	sly-mir166a v1	UCGGACCAGGCUUCAUUCCCG	21	13,19	26,00
	sly-mir166a v2	UCGGACCAGGCUUCAUUCCC**U**	21	34,77	82,40
		UCGGACCAGGCUUCAUUCCCU**U**	22	-	1,46
	sly-mir166a v4	U**U**GGACCAGGCUUCAUUCCCC	21	151,66	467,49
	sly-mir166c	UCGGACCAGGCUUCAUUCCUC	21	17373,70	16372,71
	sly-mir166c v2	UCGGACCAGGCUUCAUUCCU**A**	21	-	4,21
	sly-mir166c v3	UCGGA**U**CAGGCUUCAUUCCUC	21	3,60	2,93
	sly-mir166c v4	UCGGACCAGGCUUCAUUCCU**U**	21	4,20	14,47
	sUu-miR166d-5p	AGAAUGUCGUCUGGUUCGAGA	21	95,31	123,78
miR168	sly-miR168a,b-5p	UCGCUUGGUGCAGGUCGGGAC	21	2087,34	1739,39
	sly-miR168a,b-5p v1	UCGCUUGGUGCAG**A**UCGGGAC	21	-	0,18
	sly-miR168a,b-5p v2	UCGCUUGGUGCAGGUCGGGA**A**	21	3,00	4,39
	sly-miR168a-3p	CCUGCCUUGCAUCAACUGAAU	21	99,51	38,45
miR169	sly-miR169e-3p	UGGCAAGCAUCUUUGGCGACU	21	157,66	192,64
	sly-miR169b	UAGCCAAGGAUGACUUGCCUG	21	5,99	1,10
	sly-miR169e	UAGCCAAGGAUGACUUGCCUU	21	1,20	-
		UAGCCAAGGAUGACUUGCCUU**U**	22	-	0,37
miR172	sly-miR172a,b	AGAAUCUUGAUGAUGCUGCAU	21	9,59	4,94
	sly-miR172a,b v1	GAGAAUCUUGAUGAUGCUGCA	21	2,40	0,37
		**G**AGAAUCUUGAUGAUGCUGCAU	22	0,60	0,18
	sly-miR172a,b v3	**UG**AGAAUCUUGAUGAUGCUGC	21	0,60	0,18
	sly-miR172a,b v4	**U**GAAUCUUGAUGAUGCUGCAU	21	0,60	0,73
	sUu-miR172d-3p	GGAAUCUUGAUGAUGCUGCAG	21	1,20	-
	sUu-miR172b-5p	GCAGCACCAU**U**AAGAUUCACA	21	-	0,55
	sUu-miR172d-5p	GGAGCAUCAUCAAGAUUCACA	21	-	0,37
miR482	sly-miR482a	UUUCCAAUUCCACCCAUUCCU	21	2,40	0,92
		UUUCCAAUUCCACCCAUUCCUA	22	14,99	9,16
	sly-miR482b	UCUUGCCUACACCGCCCAUGC	21	-	0,37
		UCUUGCCUACACCGCCCAUGCC	22	868,62	1407,41
	sly-miR482d-3p	UUUCCUAUUCCACCCAUGCCA	21	2,40	0,18
		UUUCCUAUUCCACCCAUGCCAA	22	19,18	8,42
	sly-miR482e-3p	UCUUUCCUACUCCUCCCAUAC	21	-	0,92
		UCUUUCCUACUCCUCCCAUAC**C**	22	1038,87	462,36
	sly-miR482e-3p v1	CUUUCCUACUCCUCCCAUACC	22	1,20	-
miR6024	sly-miR6024	UUUUAGCAAGAGUUGUUUUAC	21	317,12	118,84
		UUUUAGCAAGAGUUGUUUUACC	22	2,40	1,28
	sly-miR6024 v1	UUUAGCAAGAGUUGUUUUACC	21	227,80	86,25

*** miRNAs were identified in our dataset by perfect matches to sequences from the miRNA repository (miRBASE release 21) and using miRPROF tool. ppm= part per million.**
