# Supplementary material for: A Short Indel-Lacking-Resistance Gene Triggers Silencing of the Photosynthetic Machinery Components Through TYLCSV-Associated Endogenous siRNAs in Tomato
Source: Front Plant Sci. 2018 Oct 11;9:1470. doi: 10.3389/fpls.2018.01470 (PMC6193080; doi:10.3389/fpls.2018.01470)
Supplement: TABLE S3 — In silico analysis of miRNA targets among the selected gene families, validated by PARE analysis. [file Table_3.PDF]

Supplementary Table S3. *In silico* analysis of miRNA targets among the selected gene families, validated by PARE analysis.

Target ID	Presence in PARE library	miRNA family by psRNATarget finder	Target start	Target end	Target Site Location (target/total exons)
Squamosa promoter binding proteins (EPlGT00820000103254)					
Solyc02g077920	✔	miR156	830	849	3' UTR
Solyc04g045560	✔	miR156	788	808	exon 4/4*
Solyc05g012040	✔	miR156	1419	1439	exon 4/5
Solyc05g015510	✔	miR156	2951	2971	exon 4/4
Solyc05g015840	✔	miR156/168	749/614	769/633	exon 3/3
Solyc10g009080	✔	miR156	797	816	3' UTR
Solyc10g078700	✔	miR156	764	784	exon 3/3*
Solyc01g068100		-	-	-	-
Solyc01g080670		miR9472	873	892	exon 2/10
Solyc01g090730		-	-	-	-
Solyc03g114850		miR156	1472	1491	3' UTR
Solyc05g053240		-	-	-	-
Solyc07g053810		-	-	-	-
Solyc07g062980		miR156	439	458	3' UTR
Solyc10g018780		-	-	-	-
Solyc12g038520		miR156	971	991	exon 3/3*
Auxin response factors (EPlGT00850000106226)					
Solyc06g075150	✔	-	-	-	-
Solyc09g007810	✔	miR160	1597	1617	exon 3/4
Solyc11g069500	✔	miR160	1303	1322	exon 2/4
Solyc05g056040		-	-	-	-
Solyc10g086130		miR160	1368	1388	exon 2/2
Solyc11g013480		-	-	-	-
Homebox-leucine zipper proteins (EPlGT00140000000795)					
Solyc02g024070	✔	miR166	809	828	boundary exons 4-5/18
Solyc03g120910	✔	miR166	849-850	869	boundary exons 4-5/18
Solyc08g066500	✔	miR390	39	58	boundary 5' UTR-exon 1/18
Solyc11g069470	✔	miR166	560-561	580	boundary exons 4-5/18
Solyc12g044410	✔	miR166	548-549	568	boundary exons 4-5/18
Solyc01g057960		-	-	-	-
Solyc02g069830		miR166	619	638	boundary exons 6-7/20
Argonautes (EPlGT00820000103293)					
Solyc03g098280**	✔	miR168	24	43	-
Solyc06g072300	✔	miR168	2107-2108	2127	exon 1/21
Solyc01g008960		-	-	-	-
Solyc01g010970		-	-	-	-
Solyc01g096750		-	-	-	-
Solyc02g069260		miR403	2108	2127	exon 3/3
Solyc02g069270		-	-	-	-
Solyc02g069280		-	-	-	-
Solyc03g111760		-	-	-	-
Solyc06g073530		-	-	-	-
Solyc06g073540		-	-	-	-
Solyc06g074730		-	-	-	-
Solyc07g049500		-	-	-	-
Solyc09g082830		-	-	-	-
Solyc12g006790		-	-	-	-

Nuclear transcription factors (HAP) (EPlGT00140000000465)					
Solyc01g006930	✔	miR169	1317	1337	3' UTR
Solyc01g087240	✔	miR169	1160	1180	3' UTR
Solyc08g062210	✔	miR169	1101	1120	3' UTR
Solyc01g008490		miR169	1058	1077	3' UTR
Solyc02g069860		-	-	-	-
Solyc03g121940	✔	miR169	1022	1041	3' UTR
Solyc10g079150		-	-	-	-
Solyc10g081840		-	-	-	-
Solyc11g065700		-	-	-	-
Solyc12g009050		-	-	-	-
Transcription factors (AP, RAP) (EPlGT00850000106232)					
Solyc02g064960	✔	miR172	1733-1735	1753-1755	exon 10/10
Solyc02g093150	✔	miR172	1561-1563	1581-1583	exon 10/10
Solyc04g049800	✔	miR172	1106-1108	1126-1128	exon 9/9
Solyc09g007260	✔	miR172	1512-1514	1532-1534	exon 9/9
Solyc11g072600	✔	miR172	1277-1278	1297-1299	exon 9/9*
Solyc03g044300		miR172	1373-1375	1393-1395	exon 9/9
Solyc06g075510		miR172	1511-1513	1531-1533	exon 10/10
Solyc10g084340		miR172	1242	1261-1262	exon 10/10*
Disease resistance proteins (EPlGT00820000103688)					
Solyc05g008070	✔	miR6024/miR482	543-544/528	563-564/549	exon 1/2*
Solyc01g017690		-	-	-	-
Solyc01g087200		miR6024/miR482	532-533/516	552-553/536	exon 2/3 / boundary exons 1-2/3
Solyc01g103610		-	-	-	-
Solyc04g007080		-	-	-	-
Solyc04g007090		-	-	-	-
Solyc04g011890		-	-	-	-
Solyc04g011960		-	-	-	-
Solyc04g011980		-	-	-	-
Solyc04g011990		-	-	-	-
Solyc04g012000		-	-	-	-
Solyc04g012010		-	-	-	-
Solyc06g062440		-	-	-	-
Solyc07g005770		-	-	-	-
Solyc07g009180		miR6024	553-554	574	exon 1/1*
Solyc07g009190		-	-	-	-
Solyc07g009200		-	-	-	-
Solyc07g049700		miR482	544	565	exon 1/1*
Solyc08g074250		-	-	-	-
Solyc10g008230		miR6024	511-512	531	exon 1/3*
Solyc10g008240		miR6024	511-512	531-532	exon 1/2*
Solyc10g051050		-	-	-	-
Solyc11g006520		miR6024/ miR482	526-527/511	546-547/532	exon 2/3
Solyc11g006530		miR482	526	547	exon 2/4*
Solyc11g006630		miR6024/ miR482	547-548/532	567-568/553	exon 2/3
Solyc11g006640		miR6024	547-548	567-568	exon 2/4
Solyc11g020080		-	-	-	-
Solyc11g020100		miR482	520	541	exon 1/3*
Solyc11g069020		miR6024	571-572	591-592	exon 1/1*
Solyc12g017730		-	-	-	-
Solyc12g017800		miR482	526	547	exon 2/4*
Solyc12g017810		-	-	-	-

*unknown UTR
**synonym of NM_001279332.2
