# Supplementary material for: A Short Indel-Lacking-Resistance Gene Triggers Silencing of the Photosynthetic Machinery Components Through TYLCSV-Associated Endogenous siRNAs in Tomato
Source: Front Plant Sci. 2018 Oct 11;9:1470. doi: 10.3389/fpls.2018.01470 (PMC6193080; doi:10.3389/fpls.2018.01470)
Supplement: TABLE S4 — Output of PAREsnp listing 5′ RNA remnants of cDNA targeted by 21-nt-long secondary siRNAs in tomato TYLCSV-infected plant tissues. [file Table_4.PDF]

Supplementary Table S4. Output of PAREsnp listing 5' RNA remnants of cDNA targeted by 21-nt-long secondary siRNAs in tomato TYLCSV-infected plant tissues.

<table>
<tr><th>Duplex</th><th>Gene</th><th>Category</th><th>Cleavage Position</th><th>P-Value</th><th>Fragment Abundance</th><th>Alignment Score</th><th>Short Read ID</th></tr>
<tr><td>5'   TGGTTATTGTGTATAGAAGTC 3'
      ||o|| ||||oo|o|o|| ||
3' TACGACTAACAACATGTGTTTT-AGGGTGTTT 5'</td><td>Solyc05g056050.2.1 genomic_reference:SL2.50ch05 gene_region:64597539-64599708 transcript_region:SL2.50ch05:64597539..64599708- go_terms:GO:0016020 functional_description:Chlorophyll a-b binding protein 6A chloroplastic (AHRD V1 ***- CB11_SOLLC)_contains Interpro domain(s)_IPR001344_Chlorophyll A-B binding protein ""</td><td>3</td><td>729</td><td>0.0</td><td>3</td><td>4.5</td><td>>Solyc05g008070</td></tr>
<tr><td>5'   CTTGGATGATGTCTGGGAGGC 3'
      |||||||| ||||||| | o
3' CGGAGAACCTAC-ACAGACCATGTTGATGTAA 5'</td><td>Solyc00g009020.2.1 genomic_reference:SL2.50ch00 gene_region:8742084-8746574 transcript_region:SL2.50ch00:8742084..8746574+ go_terms:GO:0008270,GO:0050897,GO:0005507 functional_description:Mitochondrial ATP synthase (AHRD V1 ***- B6TLY8_MAIZE)""</td><td>2</td><td>1100</td><td>0.03</td><td>2</td><td>4.5</td><td>>Solyc02g036270</td></tr>
<tr><td>5'   AGATAGCTGGAGGAGTTGGCC 3'
      |||o| ||||||o ||o|||
3' ATAGTCTGT-GACCTCTACAGCCGGAGTTTCA 5'</td><td>Solyc03g121950.2.1 genomic_reference:SL2.50ch03 gene_region:63997165-64006343 transcript_region:SL2.50ch03:63997165..64006343- functional_description:Ataxin-2 (AHRD V1 *--- D3BPE8_POLPA)_contains Interpro domain(s)_IPR009604_LsmAD domain ""</td><td>4</td><td>1688</td><td>0.03</td><td>1</td><td>3.5</td><td>>Solyc02g036270</td></tr>
<tr><td>5'   ATTTCTGGAATCGTGGAGATC   3'
      | ||||| |||||||| |||
3' CGACT-AAGACGTTAGCACCGCTATAGCCATC 5'</td><td>Solyc10g077120.1.1 evidence_code:10F1H1E1IEG genomic_reference:SL2.50ch10 gene_region:59320450-59321013 transcript_region:SL2.50ch10:59320450..59321013+ functional_description:Photosystem II core complex proteins psbY (AHRD V1 *-*- B6SR26_MAIZE)""</td><td>2</td><td>437</td><td>0.01</td><td>3</td><td>4.0</td><td>>Solyc02g036270</td></tr>
<tr><td>5'   GATGGTTGTGATTGCTACAAC 3'
      o|| |||||| ||||o||||
3' CTCGTTAGCAACAC-AACGGTGTTTATTAGCA 5'</td><td>Solyc09g072750.2.1 genomic_reference:SL2.50ch09 gene_region:60878372-60879115 transcript_region:SL2.50ch09:60878372..60879115- functional_description:Unknown Protein (AHRD V1)""</td><td>1</td><td>461</td><td>0.01</td><td>3</td><td>4.0</td><td>>Solyc02g036270</td></tr>
<tr><td>5'   TTAACATTAGAAACTCAAGTC 3'
      o||||||| |||||||| |o
3' TCTCGATTGTAAGCTTTGAGT-CGATAGTCAT 5'</td><td>Solyc03g044150.2.1 genomic_reference:SL2.50ch03 gene_region:12190436-12193226 transcript_region:SL2.50ch03:12190436..12193226- go_terms:GO:0004252 functional_description:Subtilisin-like protease (AHRD V1 ***- A9XG40_TOBAC)_contains Interpro domain(s)_IPR015500_Peptidase S8 subtilisin-related ""</td><td>2</td><td>2432</td><td>0.02</td><td>2</td><td>4.0</td><td>>Solyc02g036270</td></tr>
<tr><td>5'   CTTGGATGATGTCTGGGAGGC   3'
      ||||o||o||||| |o|| ||
3' GGGAGAACTTATTACAG-CTCTACGGAAAGAG 5'</td><td>Solyc09g005550.2.1 genomic_reference:SL2.50ch09 gene_region:369215-377428 transcript_region:SL2.50ch09:369215..377428- go_terms:GO:0004683 functional_description:Calcium dependent protein kinase 13 (AHRD V1 **** B9IGA3_POPTR)_contains Interpro domain(s)_IPR002290_Serine/threonine protein kinase ""</td><td>4</td><td>1721</td><td>0.04</td><td>1</td><td>3.5</td><td>>Solyc02g036270</td></tr>
<tr><td>5'   ACAATTCAAGCTGAGAT-AGCT   3'
      || ||o|||| ||||| |o||
3' ACTATGCTAGGTTCGTCTCTAGTTGATAGAGG 5'</td><td>Solyc04g077020.2.1 genomic_reference:SL2.50ch04 gene_region:59583881-59586708 transcript_region:SL2.50ch04:59583881..59586708+ go_terms:GO:0046982,GO:0005200 functional_description:Tubulin alpha-3 chain (AHRD V1 ***- B6SPX4_MAIZE)_contains Interpro domain(s)_IPR002452_Alpha tubulin ""</td><td>2</td><td>452</td><td>0.05</td><td>2</td><td>4.0</td><td>>Solyc02g036270</td></tr>
<tr><td>5'   AGATCATGAATTTTGCAAGTT 3'
      | |||| ||| | ||||o||
3' TATGTGTAGT-CTTTATACGTTTAACATCTGA 5'</td><td>Solyc08g015780.2.1 genomic_reference:SL2.50ch08 gene_region:5497369-5503211 transcript_region:SL2.50ch08:5497369..5503211- functional_description:F-box/ankyrin repeat protein SKIP35 (AHRD V1 ***- SKI35_ARATH)_contains Interpro domain(s)_IPR002110_Ankyrin ""</td><td>1</td><td>2467</td><td>0.03</td><td>2</td><td>4.5</td><td>>Solyc05g008070</td></tr>
<tr><td>5'   CATCATTGCTCATTTCTTGAT 3'
      | ||| ||||||||o || ||
3' CAAGGAAGTCACGAGTAAGAAAGTAGTTGGTT 5'</td><td>Solyc09g065910.1.1 evidence_code:10F1H1E1IEG genomic_reference:SL2.50ch09 gene_region:59800474-59800686 transcript_region:SL2.50ch09:59800474..59800686- go_terms:GO:0009523 functional_description:Photosystem II reaction center W protein (AHRD V1 ***- B6TMB3_MAIZE)_contains Interpro domain(s)_IPR009806_Photosystem II protein PsbW class 2 ""</td><td>3</td><td>67</td><td>0.03</td><td>3</td><td>4.5</td><td>>Solyc05g008070</td></tr>
<tr><td>5'   ATCGTGGAGATCAGTTGCGTT 3'
      | |o|o||||||||||o| ||
3' GTGCTCGTATCTCTAGTCAATGGAAGAGGAGT 5'</td><td>Solyc08g076720.2.1 genomic_reference:SL2.50ch08 gene_region:57827103-57836786 transcript_region:SL2.50ch08:57827103..57836786- go_terms:GO:0010329 functional_description:Uncharacterized ABC transporter ATP-binding protein TM_0288 (AHRD V1 *--* Y288_THEMA)_contains Interpro domain(s)_IPR003439_ABC transporter-like ""</td><td>4</td><td>2713</td><td>0.02</td><td>1</td><td>3.5</td><td>>Solyc02g036270</td></tr>
<tr><td>5'   TGAACAGAATTTGGAAAGGTT 3'
      | ||||||||||o|||| o|
3' TACCAATTGTCTTAAATCTTTGTATCAACCCT 5'</td><td>Solyc04g078070.2.1 genomic_reference:SL2.50ch04 gene_region:60508246-60511703 transcript_region:SL2.50ch04:60508246..60511703- functional_description:Expressed protein having alternate splicing products (AHRD V1 *--- Q75KB8_ORYSJ)_contains Interpro domain(s)_IPR015023_Protein of unknown function DUF1909 ""</td><td>2</td><td>699</td><td>0.02</td><td>4</td><td>4.0</td><td>>Solyc02g036270</td></tr>
<tr><td>5'   TGTAATTTGAACGGCTCTTCT   3'
      |o||| ||||||o|| ||o||
3' GTGTATATTTAACTTGTCG-GAGGAGACATTC 5'</td><td>Solyc11g044800.1.1 evidence_code:10F0H1E1IEG genomic_reference:SL2.50ch11 gene_region:36091790-36097197 transcript_region:SL2.50ch11:36091790..36097197+ go_terms:GO:0008081 functional_description:PHP domain-containing protein (AHRD V1 ***- D7L064_ARALY)_contains Interpro domain(s)_IPR003141_Polymerase and histidinol phosphatase N-terminal ""</td><td>4</td><td>762</td><td>0.03</td><td>1</td><td>3.5</td><td>>Solyc02g036270</td></tr>
<tr><td>5'   CTGAACAATATA-ATAAGAGAC 3'
      ||| |||||||| |||_|o||
3' GACAGACGTGTTATATATATGATTTGATGAGA 5'</td><td>Solyc06g073110.2.1 genomic_reference:SL2.50ch06 gene_region:41424260-41431619 transcript_region:SL2.50ch06:41424260..41431619- functional_description:Legume lectin beta domain (AHRD V1 *-*- A2Q3C0_MEDTR)_contains Interpro domain(s)_IPR010341_Protein of unknown function DUF936 plant ""</td><td>0</td><td>2791</td><td>0.02</td><td>2</td><td>4.5</td><td>>Solyc05g008070</td></tr>
<tr><td>5'   AGTACTGAGGCTTGGGACCAA 3'
      |o|||o|||||| ||| ||||
3' CTTGTTATGGCTCCGACCCCGGGTTCGGTTCC 5'</td><td>Solyc12g017910.1.1 evidence_code:10F0H1E1IEG genomic_reference:SL2.50ch12 gene_region:7757083-7763435 transcript_region:SL2.50ch12:7757083..7763435- go_terms:GO:0009674 functional_description:Potassium transporter (AHRD V1 **** Q1T722_PHRAU)_contains Interpro domain(s)_IPR018519_Potassium uptake protein kup_IPR003855_K+ potassium transporter ""</td><td>4</td><td>1593</td><td>0.01</td><td>1</td><td>3.0</td><td>>Solyc05g008070</td></tr>
<tr><td>5'   ATAACCATCAATTC-ATCATTG   3'
      |||| ||||||||| |||| ||
3' TCGATATTAGTAGTTAAGGTAGTTACCACAAC 5'</td><td>Solyc06g072380.2.1 genomic_reference:SL2.50ch06 gene_region:41035937-41046448 transcript_region:SL2.50ch06:41035937..41046448- go_terms:GO:0004371 functional_description:Dihydroxyacetone/glycerone kinase-like protein (AHRD V1 **** Q9LSH1_ARATH)_contains Interpro domain(s)_IPR012734_Dihydroxyacetone kinase ""</td><td>4</td><td>1013</td><td>0.03</td><td>1</td><td>3.0</td><td>>Solyc05g008070</td></tr>
<tr><td>5'   TGAACAGAATTTGGAAAGGTT   3'
      |o|||| |||||o |||| ||
3' GGTTATTTGTATTAAATATTTC-AACTCCGTT 5'</td><td>Solyc10g039290.1.1 evidence_code:10F0H1E1IEG genomic_reference:SL2.50ch10 gene_region:20937256-20939892 transcript_region:SL2.50ch10:20937256..20939892- go_terms:GO:0042936 functional_description:Peptide transporter-like protein (AHRD V1 **-* Q9LSE8_ARATH)_contains Interpro domain(s)_IPR000109_TGF-beta receptor type I/II extracellular region ""</td><td>0</td><td>1586</td><td>0.01</td><td>3</td><td>4.0</td><td>>Solyc02g036270</td></tr>
<tr><td>5'   CGATCTTTGAGTGTACAAGAC   3'
       || |||||| ||o||||o||
3' TCTGTCTTGAAACT-ACGTGTTTTGAGTTAGT 5'</td><td>Solyc05g050200.2.1 genomic_reference:SL2.50ch05 gene_region:59394632-59396589 transcript_region:SL2.50ch05:59394632..59396589+ go_terms:GO:0003743 functional_description:Eukaryotic translation initiation factor 1A (AHRD V1 **** Q7Y1V3_ORYSJ)_contains Interpro domain(s)_IPR001253_Translation initiation factor 1A (eIF-1A) ""</td><td>2</td><td>689</td><td>0.01</td><td>2</td><td>4.0</td><td>>Solyc05g008070</td></tr>
<tr><td>5'   GCCTGAGTGGATATGAGGACT   3'
      | ||||||||||||o||_ |
3' GGATCTGACTCACCTATATTCGCTATTATCGA 5'</td><td>Solyc08g061610.2.1 genomic_reference:SL2.50ch08 gene_region:46389278-46410833 transcript_region:SL2.50ch08:46389278..46410833- go_terms:GO:0004008 functional_description:Copper-exporting P-type ATPase A (AHRD V1 **** COPA_STAES)_contains Interpro domain(s)_IPR006403_ATPase P type cation/copper-transporter ""</td><td>2</td><td>1056</td><td>0.05</td><td>2</td><td>4.5</td><td>>Solyc02g036270</td></tr>
</table>

<table>
<tr><th></th><th></th><th></th><th></th><th></th><th></th><th></th><th></th></tr>
<tr><td>5' GAATCATCACACGTCTACGAT 3'
o|| ||| ||||oo|||||||
3' ATTATTTTGTATTGTGTGGATGCTAATCGTTT 5'</td><td>Solyc07g047670.2.1 genomic_reference:SL2.50ch07 gene_region:56216949-56224432 transcript_region:SL2.50ch07:56216949..56224432+ go_terms:GO:0003713 functional_description:Pescadillo homolog 1 (AHRD V1 ***- B2RDF2_HUMAN)_contains Interpro domain(s)_IPR010613_Pescadillo N-terminal ""</td><td>4</td><td>1948</td><td>0.03</td><td>1</td><td>3.5</td><td>>Solyc05g008070</td></tr>
<tr><td>5' TGAA-CAAACTTGGAATTCCTA 3'
|||| |||||| ||_||||o|
3' CGTACTTGGTTTGATCCCAAAGGGTTCATCAG 5'</td><td>Solyc03g005760.1.1 evidence_code:10F0H1E1IEG genomic_reference:SL2.50ch03 gene_region:538196-538999 transcript_region:SL2.50ch03:538196..538999- go_terms:GO:0005515,GO:0016168 functional_description:Chlorophyll a-b binding protein 3C-like (AHRD V1 **** Q2XTE0_SOLTU)_contains Interpro domain(s)_IPR001344_Chlorophyll A-B binding protein ""</td><td>2</td><td>454</td><td>0.04</td><td>15</td><td>4.5</td><td>>Solyc02g036270</td></tr>
<tr><td>5' TTGATTGTTGACTCCACTAAT 3'
|| | ||||| |||||||| |
3' TTTCAAGT-ACAACCGAGGTGATCAATAACGA 5'</td><td>Solyc10g083760.1.1 evidence_code:10F1H1E1IEG genomic_reference:SL2.50ch10 gene_region:62837524-62842849 transcript_region:SL2.50ch10:62837524..62842849- go_terms:GO:0008152,GO:0006520,GO:0009097 functional_description:Threonine dehydratase biosynthetic (AHRD V1 ***- D9SD66_9PROT)_contains Interpro domain(s)_IPR000634_Serine/threonine dehydratase pyridoxal-phosphate-binding site_IPR005787_Threonine dehydratase I ""</td><td>1</td><td>1230</td><td>0.0</td><td>2</td><td>4.0</td><td>>Solyc05g008070</td></tr>
<tr><td>5' CTTATAATCTTGGATGATGTC 3'
||| |||o|||o|| | |o||
3' AAACGAAGATTGGAATCTCC-ATAGCCCTGTG 5'</td><td>Solyc06g005360.2.1 genomic_reference:SL2.50ch06 gene_region:372206-374775 transcript_region:SL2.50ch06:372206..374775+ go_terms:GO:0051015 functional_description:Actin depolymerizing factor 3 (AHRD V1 **** A1XJ46_GOSHI)_contains Interpro domain(s)_IPR002108_Actin-binding cofilin/tropomyosin type ""</td><td>2</td><td>281</td><td>0.04</td><td>2</td><td>4.5</td><td>>Solyc02g036270</td></tr>
<tr><td>5' TTCTCTTATGCACAACTGCCT 3'
o| ||| |||||| ||oo|||
3' GAAGGA-AGACTACGTGGTGGTGGAGAACTCA 5'</td><td>Solyc10g051380.1.1 evidence_code:10F0H1E0IEG genomic_reference:SL2.50ch10 gene_region:47515222-47515986 transcript_region:SL2.50ch10:47515222..47515986+ go_terms:GO:0008266 functional_description:Glycine-rich RNA-binding protein (AHRD V1 **** B2YKT9_TOBAC)_contains Interpro domain(s)_IPR015465_RNA recognition motif glycine rich protein ""</td><td>1</td><td>368</td><td>0.04</td><td>2</td><td>4.5</td><td>>Solyc05g008070</td></tr>
<tr><td>5' TGGTGACCCATGCATGTTTCT 3'
|o|| |||||||| || ||||
3' AGTTATCA-TGGGTACGGACCAAGACCTAGTC 5'</td><td>Solyc08g074630.1.1 evidence_code:10F1H1E1IEG genomic_reference:SL2.50ch08 gene_region:55921673-55923436 transcript_region:SL2.50ch08:55921673..55923436+ go_terms:GO:0008152,GO:0055114 functional_description:Polyphenol oxidase (AHRD V1 ***- Q41428_SOLTU)_contains Interpro domain(s)_IPR016213_Polyphenol oxidase plant ""</td><td>4</td><td>959</td><td>0.05</td><td>1</td><td>3.5</td><td>>Solyc05g008070</td></tr>
<tr><td>5' TGTAATTTGAACGGCTCTTCT 3'
|||||o|||| |o|||| ||
3' TGCAACATTAGACTTCCTGAGA-GAGAAAGCC 5'</td><td>Solyc11g068820.1.1 evidence_code:10F0H1E1IEG genomic_reference:SL2.50ch11 gene_region:50513890-50516507 transcript_region:SL2.50ch11:50513890..50516507- go_terms:GO:0005840 functional_description:50S ribosomal protein L27 (AHRD V1 ***- Q8LEF5_ARATH)_contains Interpro domain(s)_IPR001684_Ribosomal protein L27 ""</td><td>4</td><td>506</td><td>0.01</td><td>1</td><td>3.0</td><td>>Solyc02g036270</td></tr>
<tr><td>5' AATTCATGATCTTCTTAGGCA 3'
|| |||||||||||o o|||
3' CAACTT-AGTACTAGAAGAGATCGTAGGGATG 5'</td><td>Solyc03g006100.2.1 genomic_reference:SL2.50ch03 gene_region:771603-775358 transcript_region:SL2.50ch03:771603..775358+ go_terms:GO:0005515,GO:0019199 functional_description:Receptor like kinase RLK""</td><td>4</td><td>2686</td><td>0.02</td><td>1</td><td>3.0</td><td>>Solyc05g008070</td></tr>
<tr><td>5' TCAAG-AAATGAGCAATGATGA 3'
o|||| || || |||||||||
3' CTAGGTTCGTTGACACGTTACTACATGTTTTA 5'</td><td>Solyc06g074820.2.1 genomic_reference:SL2.50ch06 gene_region:42747475-42749825 transcript_region:SL2.50ch06:42747475..42749825+ go_terms:GO:0015250 functional_description:Aquaporin-like protein (AHRD V1 ***- D1M6Z5_CAPAN)_contains Interpro domain(s)_IPR012269_Aquaporin ""</td><td>3</td><td>444</td><td>0.01</td><td>2</td><td>4.5</td><td>>Solyc05g008070</td></tr>
<tr><td>5' TCTAATCGTCATAGATGATCT 3'
|o||||o|||||||| |o |
3' AAAGTGGTTAGTAGTATCTAGTGTAGAGATCC 5'</td><td>Solyc01g108500.2.1 genomic_reference:SL2.50ch01 gene_region:87565501-87571455 transcript_region:SL2.50ch01:87565501..87571455- go_terms:GO:0005515 functional_description:Polyadenylate-binding protein (AHRD V1 **-* PABP_DROME)_contains Interpro domain(s)_IPR012677_Nucleotide-binding alpha-beta plait ""</td><td>0</td><td>1606</td><td>0.03</td><td>11</td><td>4.5</td><td>>Solyc05g008070</td></tr>
<tr><td>5' CTAATCGTCATAGATGATCTT 3'
|o||||o|||||||| |o |o
3' AAGTGGTTAGTAGTATCTAGTGTAGAGATCCA 5'</td><td>Solyc01g108500.2.1 genomic_reference:SL2.50ch01 gene_region:87565501-87571455 transcript_region:SL2.50ch01:87565501..87571455- go_terms:GO:0005515 functional_description:Polyadenylate-binding protein (AHRD V1 **-* PABP_DROME)_contains Interpro domain(s)_IPR012677_Nucleotide-binding alpha-beta plait ""</td><td>2</td><td>1605</td><td>0.04</td><td>5</td><td>4.0</td><td>>Solyc05g008070</td></tr>
<tr><td>5' GAAATGAGCAATGATGAATTG 3'
|||||o|||||||||o|| ||
3' CCGTCTTTATTCGTTACTATTTCACTTGGTTT 5'</td><td>Solyc08g082020.2.1 genomic_reference:SL2.50ch08 gene_region:62100535-62107680 transcript_region:SL2.50ch08:62100535..62107680- go_terms:GO:0015228 functional_description:Mitochondrial carrier-like protein (AHRD V1 ***- Q2PYY0_SOLTU)_contains Interpro domain(s)_IPR002067_Mitochondrial carrier protein ""</td><td>4</td><td>1516</td><td>0.0</td><td>1</td><td>2.0</td><td>>Solyc05g008070</td></tr>
<tr><td>5' ACCTCAAGAAGCATCTGTCTC 3'
|||o||||||||| ||o| ||
3' ATAGTGGGGTTCTTCGTTGATATAGCCCAAAA 5'</td><td>Solyc07g039200.2.1 genomic_reference:SL2.50ch07 gene_region:42919344-42923581 transcript_region:SL2.50ch07:42919344..42923581+ go_terms:GO:0042393 functional_description:Guanine nucleotide-binding protein subunit beta (AHRD V1 *--* GBB_ARATH)_contains Interpro domain(s)_IPR017986_WD40 repeat</td><td>4</td><td>525</td><td>0.02</td><td>1</td><td>3.0</td><td>>Solyc05g008070</td></tr>
<tr><td>5' GAAGTTGTCATGGTCACTGTC 3'
||| ||| |||||o||| |||
3' CCTACTTGAACTGTACCGGTG-CAGGTACGAA 5'</td><td>Solyc06g007470.2.1 genomic_reference:SL2.50ch06 gene_region:1479427-1482088 transcript_region:SL2.50ch06:1479427..1482088- go_terms:GO:0005840 functional_description:40S ribosomal protein S26 (AHRD V1 ***- B6TXN6_MAIZE)_contains Interpro domain(s)_IPR000892_Ribosomal protein S26e ""</td><td>4</td><td>143</td><td>0.05</td><td>1</td><td>3.5</td><td>>Solyc02g036270</td></tr>
<tr><td>5' TCATATCCACTCAGGCTTAAG 3'
| |o|||| | |||||||||
3' CACTATTGTAGGGGTGTCCGAATTATAATGTA 5'</td><td>Solyc01g108910.2.1 genomic_reference:SL2.50ch01 gene_region:87797493-87799653 transcript_region:SL2.50ch01:87797493..87799653- functional_description:COSII_At2g15890 (Fragment) (AHRD V1 *-*- C0KFD9_9SOLN)""</td><td>1</td><td>769</td><td>0.02</td><td>5</td><td>4.5</td><td>>Solyc02g036270</td></tr>
<tr><td>5' TGGTTATTGTGTATAGAAGTC 3'
||o|| ||||oo|o|o|| ||
3' TACGACTAACAACATGTGTTTT-AGGGTGTTT 5'</td><td>Solyc05g056070.2.1 genomic_reference:SL2.50ch05 gene_region:64606977-64608378 transcript_region:SL2.50ch05:64606977..64608378+ go_terms:GO:0016020 functional_description:Chlorophyll a-b binding protein 6A chloroplastic (AHRD V1 ***- CB11_SOLLC)_contains Interpro domain(s)_IPR001344_Chlorophyll A-B binding protein ""</td><td>3</td><td>690</td><td>0.0</td><td>3</td><td>4.5</td><td>>Solyc05g008070</td></tr>
<tr><td>5' CGTGTGATGATTCCTTTTCTG 3'
|||||||| ||| ||o|||o
3' GCAGGCACACTAGTAAAGAGAAGGAAGAAGAG 5'</td><td>Solyc04g016590.2.1 genomic_reference:SL2.50ch04 gene_region:7438209-7447119 transcript_region:SL2.50ch04:7438209..7447119- functional_description:Unknown Protein (AHRD V1)""</td><td>2</td><td>516</td><td>0.03</td><td>2</td><td>4.0</td><td>>Solyc05g008070</td></tr>
<tr><td>5' ATTCATCATTGCTCATTTCTT 3'
||||||||||o | ||||||o
3' TGGCTAAGTAGTAATAATTAAAGAGTATTGAA 5'</td><td>Solyc02g063150.2.1 genomic_reference:SL2.50ch02 gene_region:29802717-29803963 transcript_region:SL2.50ch02:29802717..29803963+ go_terms:GO:0005515,GO:0005507 functional_description:Ribulose bisphosphate carboxylase small chain (AHRD V1 ***- Q84QE5_TOBAC)_contains Interpro domain(s)_IPR000894_Ribulose bisphosphate carboxylase small chain ""</td><td>2</td><td>765</td><td>0.02</td><td>12</td><td>3.0</td><td>>Solyc05g008070</td></tr>
<tr><td>5' CTGAACAATATAATAAGAGAC 3'
|||||||||o|||||||o||
3' GTTCAACTTGTTATGTTATTCTTTGAGAACTT 5'</td><td>Solyc06g060400.2.1 genomic_reference:SL2.50ch06 gene_region:34803093-34805179 transcript_region:SL2.50ch06:34803093..34805179- go_terms:GO:0005840 functional_description:Ribosomal protein L15 (AHRD V1 ***- B9GPA1_POPTR)_contains Interpro domain(s)_IPR000439_Ribosomal protein L15e ""</td><td>2</td><td>713</td><td>0.0</td><td>2</td><td>2.0</td><td>>Solyc05g008070</td></tr>
</table>

<table>
<tr><th></th><th></th><th></th><th></th><th></th><th></th><th></th><th></th></tr>
<tr><td>5' AACAAT-CAAGAAATGAGCAAT 3'
||| || |||||||| ||||||
3' GGTTTGGTACGTTCTTTAGTCGTTAGAGTCAG 5'</td><td>Solyc11g065730.1.1 evidence_code:10F0H1E1IEG genomic_reference:SL2.50ch11 gene_region:48394251-48402079 transcript_region:SL2.50ch11:48394251..48402079- go_terms:GO:0003676,GO:0000166 functional_description:Heterogeneous nuclear ribonucleoprotein H1 (AHRD V1 **-- Q6P0V0_DANRE)_contains Interpro domain(s)_IPR000504_RNA recognition motif RNP-1 ""</td><td>4</td><td>243</td><td>0.05</td><td>1</td><td>3.0</td><td>>Solyc05g008070</td></tr>
<tr><td>5' TGTCCTGACCCATTAACCTTG 3'
||| |||| |||||||||||
3' GCTGACAAGACTAGGTAATTGGAACATGCGTC 5'</td><td>Solyc04g005540.2.1 genomic_reference:SL2.50ch04 gene_region:355454-361509 transcript_region:SL2.50ch04:355454..361509+ go_terms:GO:0006952,GO:0006915 functional_description:Cc-nbs-lrr resistance protein""</td><td>4</td><td>848</td><td>0.0</td><td>1</td><td>2.0</td><td>>Solyc02g036270</td></tr>
<tr><td>5' GTTTGTTCAGATCAAGAGCCT 3'
|||| ||||||| ||||| ||
3' TCTTCAAAGAAGTCTACTTCTCCGAAATTGGA 5'</td><td>Solyc10g011770.2.1 genomic_reference:SL2.50ch10 gene_region:4007667-4016776 transcript_region:SL2.50ch10:4007667..4016776+ functional_description:Threonine endopeptidase (AHRD V1 **-- B6TCN7_MAIZE)""</td><td>4</td><td>323</td><td>0.04</td><td>1</td><td>3.0</td><td>>Solyc02g036270</td></tr>
<tr><td>5' ATCTTTGGAGTACTGAGGCTT 3'
o||||o||o||o||| |||
3' CTACCGGAAACTTCGTGGCTC-GAACTAGGAG 5'</td><td>Solyc02g080590.2.1 genomic_reference:SL2.50ch02 gene_region:39366516-39369239 transcript_region:SL2.50ch02:39366516..39369239+ go_terms:GO:0008017 functional_description:Autophagy-related protein 8 (AHRD V1 **-- C1EJ88_9CHLO)_contains Interpro domain(s)_IPR004241_Light chain 3 (LC3) ""</td><td>1</td><td>395</td><td>0.01</td><td>2</td><td>4.0</td><td>>Solyc05g008070</td></tr>
<tr><td>5' TGGTTATTGTGTATAGAAGTC 3'
||o|| ||||oo|o|o|| ||
3' TACGACTAACAACATGTGTTTT-AGGGTGTTT 5'</td><td>Solyc05g056060.2.1 genomic_reference:SL2.50ch05 gene_region:64601085-64608155 transcript_region:SL2.50ch05:64601085..64608155+ functional_description:Ubiquitin-like protein (Fragment) (AHRD V1 *-*- A6N0B7_ORYSI)_contains Interpro domain(s)_IPR000626_Ubiquitin ""</td><td>3</td><td>2276</td><td>0.0</td><td>3</td><td>4.5</td><td>>Solyc05g008070</td></tr>
<tr><td>5' ATCTGAACAATATAATAAGAG 3'
| ||||||||o|||||||o
3' AGTTCA-ACTTGTTATGTTATTCTTTGAGAAC 5'</td><td>Solyc06g060400.2.1 genomic_reference:SL2.50ch06 gene_region:34803093-34805179 transcript_region:SL2.50ch06:34803093..34805179- go_terms:GO:0005840 functional_description:Ribosomal protein L15 (AHRD V1 ***- B9GPA1_POPTR)_contains Interpro domain(s)_IPR000439_Ribosomal protein L15e ""</td><td>4</td><td>715</td><td>0.04</td><td>1</td><td>3.0</td><td>>Solyc05g008070</td></tr>
<tr><td>5' CTTGATCTGAACAAACTTGGA 3'
|||||||| | ||| ||| o|
3' GTACGAACTAGAGTCGTTGGAAATTAGAGAGT 5'</td><td>Solyc01g105030.2.1 genomic_reference:SL2.50ch01 gene_region:85087662-85089049 transcript_region:SL2.50ch01:85087662..85089049+ go_terms:GO:0016168 functional_description:Chlorophyll a-b binding protein chloroplastic (AHRD V1 **** CB12_PETHY)_contains Interpro domain(s)_IPR001344_Chlorophyll A-B binding protein ""</td><td>2</td><td>841</td><td>0.05</td><td>16</td><td>4.5</td><td>>Solyc02g036270</td></tr>
<tr><td>5' TCCTGACCCATTAACCTTGAA 3'
|||| ||| ||o||||||o|
3' TTGTAGGA-TGGATAGTTGGAATTGAGCTGCA 5'</td><td>Solyc08g006500.2.1 genomic_reference:SL2.50ch08 gene_region:1096757-1106781 transcript_region:SL2.50ch08:1096757..1106781+ go_terms:GO:0005217,GO:0005515 functional_description:Glutamate-gated kainate-type ion channel receptor subunit GluR5 (AHRD V1 **** B9HB97_POPTR)_contains Interpro domain(s)_IPR017103_Ionotropic glutamate-like receptor</td><td>0</td><td>3036</td><td>0.0</td><td>2</td><td>4.0</td><td>>Solyc02g036270</td></tr>
<tr><td>5' TTGACAATAACTGAACAGAAT 3'
|||| ||||||| |||||||
3' TTCTCACTG-TATTGACGTGTCTTAGACTTAC 5'</td><td>Solyc06g010030.2.1 genomic_reference:SL2.50ch06 gene_region:4786764-4792828 transcript_region:SL2.50ch06:4786764..4792828+ go_terms:GO:0016021 functional_description:MLO-like protein 3 (AHRD V1 ***- C6EWE6_VITVI)_contains Interpro domain(s)_IPR004326_Mlo-related protein ""</td><td>4</td><td>1263</td><td>0.03</td><td>1</td><td>3.0</td><td>>Solyc02g036270</td></tr>
<tr><td>5' GTCTGGGAGGCTCTTGATCTG 3'
||| |o|||| |||oo|| ||
3' ACACCAGTCTCTCCAAGAGTTA-ACCTGGGAA 5'</td><td>Solyc07g047850.2.1 genomic_reference:SL2.50ch07 gene_region:56404445-56405833 transcript_region:SL2.50ch07:56404445..56405833- go_terms:GO:0016168 functional_description:Chlorophyll a-b binding protein 4 chloroplastic (AHRD V1 **** CB24_SOLLC)_contains Interpro domain(s)_IPR001344_Chlorophyll A-B binding protein ""</td><td>2</td><td>852</td><td>0.04</td><td>15</td><td>4.5</td><td>>Solyc02g036270</td></tr>
<tr><td>5' GTTTGTTCAGATCAAGAGCCT 3'
| |||o||||||||||| ||o
3' CTGTCTAACGAGTCTAGTTCT-GGGTTACAAA 5'</td><td>Solyc01g095670.2.1 genomic_reference:SL2.50ch01 gene_region:78628729-78632811 transcript_region:SL2.50ch01:78628729..78632811- go_terms:GO:0005515 functional_description:Rhodanese-related sulfurtransferase (AHRD V1 *--- A0NUG1_9RHOB)_contains Interpro domain(s)_IPR001763_Rhodanese-like ""</td><td>4</td><td>878</td><td>0.02</td><td>1</td><td>3.0</td><td>>Solyc02g036270</td></tr>
<tr><td>5' GTAAGATTAGAACTCGCAGTT 3'
|||| |o||||| ||o |o||
3' TACTCATTGTGATCTTAAGTTTTAATGTTGTA 5'</td><td>Solyc07g007120.2.1 genomic_reference:SL2.50ch07 gene_region:1876090-1882613 transcript_region:SL2.50ch07:1876090..1882613+ go_terms:GO:0005515 functional_description:Homeobox protein knotted-1-like 3 (AHRD V1 *-*- B6T3D1_MAIZE)_contains Interpro domain(s)_IPR005541_KNOX2 ""</td><td>2</td><td>1782</td><td>0.04</td><td>2</td><td>4.5</td><td>>Solyc05g008070</td></tr>
<tr><td>5' TTCGCATTTGGTCCCAAGCCT 3'
|| ||||||| | |||| |||
3' GAATAATCGTAAACAACGGTTAGGACTTTCGA 5'</td><td>Solyc09g025270.2.1 genomic_reference:SL2.50ch09 gene_region:19293483-19298869 transcript_region:SL2.50ch09:19293483..19298869+ go_terms:GO:0005488,GO:0000151 functional_description:U-box domain-containing protein 3 (AHRD V1 **-- PUB3_ARATH)_contains Interpro domain(s)_IPR011989_Armadillo-like helical ""</td><td>4</td><td>2741</td><td>0.05</td><td>1</td><td>4.0</td><td>>Solyc05g008070</td></tr>
<tr><td>5' ACTGATGGTTGTGATTGCTAC 3'
| |||| |||||||| o|o||
3' GCGCT-ACTAACAACACTACTGGTGTGAACCC 5'</td><td>Solyc12g094620.1.1 evidence_code:10F0H1E1IEG genomic_reference:SL2.50ch12 gene_region:63142846-63144894 transcript_region:SL2.50ch12:63142846..63144894+ go_terms:GO:0055114 functional_description:Catalase (AHRD V1 ***- Q2PYW5_SOLTU)_ontains Interpro domain(s)_IPR018028_Catalase related subgroup ""</td><td>2</td><td>1120</td><td>0.01</td><td>30</td><td>30.0</td><td>>Solyc02g036270</td></tr>
<tr><td>5' TTCCCTTATAGTGATCTTGTT 3'
|||| |o||||| |||o|| |
3' ATGGAAGGTAGTATCA-TAGGACCACTGAACA 5'</td><td>Solyc03g034220.2.1 genomic_reference:SL2.50ch03 gene_region:10202373-10205106 transcript_region:SL2.50ch03:10202373..10205106+ go_terms:GO:0005515,GO:0005507 functional_description:Ribulose bisphosphate carboxylase small chain (AHRD V1 ***- A9YTZ7_SOLTU)_ontains Interpro domain(s)_IPR000894_Ribulose bisphosphate carboxylase small chain ""</td><td>2</td><td>412</td><td>0.01</td><td>22</td><td>22.0</td><td>>Solyc05g008070</td></tr>
<tr><td>5' CATCATCCA-AGATTATAAGGA 3'
||||||||| | || |||||||
3' CAAGTAGTAGGTGTATATTATTCCTGTATTTA 5'</td><td>Solyc02g089090.2.1 genomic_reference:SL2.50ch02 gene_region:45568082-45571263 transcript_region:SL2.50ch02:45568082..45571263+ go_terms:GO:0004672 functional_description:Pto-like Serine/threonine kinase protein resistance protein</td><td>4</td><td>2865</td><td>0.05</td><td>1</td><td>1.0</td><td>>Solyc02g036270</td></tr>
</table>
