# Supplementary material for: A Short Indel-Lacking-Resistance Gene Triggers Silencing of the Photosynthetic Machinery Components Through TYLCSV-Associated Endogenous siRNAs in Tomato
Source: Front Plant Sci. 2018 Oct 11;9:1470. doi: 10.3389/fpls.2018.01470 (PMC6193080; doi:10.3389/fpls.2018.01470)
Supplement: TABLE S5 — Output of PAREsnp listing 5′ RNA remnants of cDNA targeted by 22-nt-long secondary siRNAs in tomato TYLCSV-infected plant tissues. [file Table_5.PDF]

Supplementary Table S5. Output of PAREsnp listing 5' RNA remnants of cDNA targeted by 22-nt-long secondary siRNAs in tomato TYLCSV-infected plant tissues.

<table>
<tr><th>Duplex</th><th>Gene</th><th>Category</th><th>Cleavage Position</th><th>P-Value</th><th>Fragment Abundance</th><th>Short Read ID</th></tr>
<tr><td>5' TGAACAAGAAAATGGTTA-ATGG 3'
| ||||| ||||||o||| ||oo
3' ACTCAGTTGTTATTTTACTAATGTATTGTTTA 5'</td><td>Solyc05g005560.2.1 genomic_reference:SL2.50ch05 gene_region:402750-405238 transcript_region:SL2.50ch05:402750..405238+ go_terms:GO:0004650 functional_description:BURP domain-containing protein (Fragment) (AHRD V1 *--- C1PI44_ARATH)_contains Interpro domain(s)_IPR004873_BURP ""</td><td>2</td><td>2169</td><td>0.03</td><td>4</td><td>>NM_00127933_AGO1B_12</td></tr>
<tr><td>5' GGAGATTCGTGTATCGAAGATG 3'
|||||| ||o||||o||o|oo
3' ACTCACTCTAAACATATAGTTTTTGTCTATGG 5'</td><td>Solyc02g077740.2.1 genomic_reference:SL2.50ch02 gene_region:37189312-37191744 transcript_region:SL2.50ch02:37189312..37191744-functional_description:OB-fold nucleic acid binding domain containing protein (AHRD V1 ***- B6SHT0_MAIZE)_contains Interpro domain(s)_IPR012340_Nucleic acid-binding</td><td>1</td><td>704</td><td>0.0</td><td>2</td><td>>Solyc05g008070.2.1_296</td></tr>
<tr><td>5' TTTGCA-CTGACGCTGTTGAGGG 3'
| |||| |||| |||| ||||o|
3' TAGACACGTCGACTCCGACTACTCTCGTTAGT 5'</td><td>Solyc07g064150.2.1 genomic_reference:SL2.50ch07 gene_region:63638382-63639895 transcript_region:SL2.50ch07:63638382..63639895+ go_terms:GO:0005515,GO:0003743 functional_description:Translation initiation factor SUI1 (AHRD V1 **** A4QR10_MAGGR)_contains Interpro domain(s)_IPR005874_Eukaryotic translation initiation factor SUI1 ""</td><td>2</td><td>419</td><td>0.02</td><td>4</td><td>>Solyc02g036270.2.1_200</td></tr>
<tr><td>5' AGATAGCTGGAGGAGTTGGCCT 3'
|||o| ||||||o ||o|||||
3' ATAGTCTGT-GACCTCTACAGCCGGAGTTTCA 5'</td><td>Solyc03g121950.2.1 genomic_reference:SL2.50ch03 gene_region:63997165-64006343 transcript_region:SL2.50ch03:63997165..64006343-functional_description:Ataxin-2 (AHRD V1 *--- D3BPE8_POLPA)_contains Interpro domain(s)_IPR009604_LsmAD domain ""</td><td>4</td><td>1688</td><td>0.0</td><td>1</td><td>>Solyc02g036270.2.1_36</td></tr>
<tr><td>5' AAAGTAACATTGACAACGCGAC 3'
| ||||| ||o||||||| ||
3' AGAATGTCATTCTAGCTGTTGC-CTCTGTAGT 5'</td><td>Solyc05g014470.2.1 genomic_reference:SL2.50ch05 gene_region:8322381-8324881 transcript_region:SL2.50ch05:8322381..8324881+ go_terms:GO:0004365 functional_description:Glyceraldehyde 3-phosphate dehydrogenase (AHRD V1 **** Q8LK04_SOLTU)_contains Interpro domain(s)_IPR000173_Glyceraldehyde 3-phosphate dehydrogenase ""</td><td>2</td><td>840</td><td>0.03</td><td>4</td><td>>Solyc02g036270.2.1_82</td></tr>
<tr><td>5' TGACAACAGTCCATGCTTTCTG 3'
|||||||||| |||||||oo
3' TAGGTGTGTTGTCAGGAACGAAAGGTATCAAC 5'</td><td>Solyc02g069460.2.1 genomic_reference:SL2.50ch02 gene_region:33917839-33919502 transcript_region:SL2.50ch02:33917839..33919502-go_terms:GO:0009538 functional_description:Photosystem I reaction center subunit III (AHRD V1 ***- Q9XQB4_PHAAU)_contains Interpro domain(s)_IPR003666_Photosystem I reaction centre protein PsaF subunit III ""</td><td>2</td><td>671</td><td>0.01</td><td>7</td><td>>Solyc05g008070.2.1_379</td></tr>
<tr><td>5' TTTCCAACTGATCATAGCTTAG 3'
|o|||||||||| ||| ||| |
3' ACCCAGAGGTTGACTAATAT-GAACCTTTACA 5'</td><td>Solyc07g005600.2.1 genomic_reference:SL2.50ch07 gene_region:487667-492520 transcript_region:SL2.50ch07:487667..492520-go_terms:GO:0005515,GO:0004888 functional_description:Transmembrane 9 superfamily protein member 4 (AHRD V1 **** B6SXZ2_MAIZE)_contains Interpro domain(s)_IPR004240_Nonaspanin (TM9SF) ""</td><td>4</td><td>1075</td><td>0.02</td><td>1</td><td>>Solyc02g036270.2.1_130</td></tr>
<tr><td>5' TTGTTTCACAAACATCTCGGAG 3'
o |||||| | |||o||||||o
3' GGGTGTCAAAGT-TATGTGGAGCCTTTTTCTT 5'</td><td>Solyc06g082140.2.1 genomic_reference:SL2.50ch06 gene_region:44340967-44341735 transcript_region:SL2.50ch06:44340967..44341735-go_terms:GO:0005840 functional_description:Unknown Protein (AHRD V1)_contains Interpro domain(s)_IPR007836_Ribosomal protein L41 ""</td><td>2</td><td>443</td><td>0.05</td><td>3</td><td>>Solyc02g036270.2.1_92</td></tr>
<tr><td>5' TTGGTCCCAAGCCTCAGTACTC 3'
|||o|| ||| ||| |||||o|
3' GAAGAACTAGAGTT-GGACTCATGGGTAGTAC 5'</td><td>Solyc07g045240.2.1 genomic_reference:SL2.50ch07 gene_region:55694997-55697651 transcript_region:SL2.50ch07:55694997..55697651+ go_terms:GO:0008266 functional_description:RNA-binding protein-like (AHRD V1 *--* Q8L779_ARATH)_contains Interpro domain(s)_IPR012677_Nucleotide-binding alpha-beta plait ""</td><td>4</td><td>521</td><td>0.05</td><td>1</td><td>>Solyc05g008070.2.1_235</td></tr>
<tr><td>5' TTGTTTCACAAACATCTCGGAG 3'
|| ||||||| ||| ||| ||o
3' GACCAA-AAAGTGTATGTCGAGACTTTACATA 5'</td><td>Solyc06g063370.2.1 genomic_reference:SL2.50ch06 gene_region:36437291-36439451 transcript_region:SL2.50ch06:36437291..36439451-go_terms:GO:0016020 functional_description:Chlorophyll a-b binding protein 1A chloroplastic (AHRD V1 ***- CB2A_PYRPY)_contains Interpro domain(s)_IPR001344_Chlorophyll A-B binding protein ""</td><td>3</td><td>1077</td><td>0.0</td><td>3</td><td>>Solyc02g036270.2.1_92</td></tr>
<tr><td>5' GAAGCAGCAGTAGGATTTGGGC 3'
o||| |||||| |||o|||o|
3' TCCCTTTCTTCGTCA-CCTGAACTCAGGTCAC 5'</td><td>Solyc02g085950.2.1 genomic_reference:SL2.50ch02 gene_region:43293286-43294312 transcript_region:SL2.50ch02:43293286..43294312+ go_terms:GO:0005515,GO:0005507 functional_description:Ribulose bisphosphate carboxylase small chain (AHRD V1 ***- A9YTZ7_SOLTU)_contains Interpro domain(s)_IPR000894_Ribulose bisphosphate carboxylase small chain ""</td><td>3</td><td>132</td><td>0.0</td><td>3</td><td>>Solyc11g069470.1.1 _8</td></tr>
<tr><td>5' CTGACAGTGACCATGACAACTT 3'
||||||o||| ||o| |||||
3' ATAAGACTGTTACTTTTATTTTTGAAATGTAA 5'</td><td>Solyc02g086840.2.1 genomic_reference:SL2.50ch02 gene_region:44022590-44025483 transcript_region:SL2.50ch02:44022590..44025483-go_terms:GO:0005975,GO:0005488 functional_description:Kinesin light chain-like protein (AHRD V1 ***- D2DWB2_PHAVU)_contains Interpro domain(s)_IPR011990_Tetratricopeptide-like helical ""</td><td>4</td><td>2382</td><td>0.05</td><td>1</td><td>>Solyc02g036270.2.1_23</td></tr>
<tr><td>5' TTAGAAAAGACACCTCGCGAGG 3'
|o|| |||||||| || |o||o
3' CTCGAGTCCTTTCTGTGTAG-GTTCTCGTGGT 5'</td><td>Solyc02g071010.1.1 evidence_code:10F0H1E1IEG genomic_reference:SL2.50ch02 gene_region:35100071-35100868 transcript_region:SL2.50ch02:35100071..35100868+ go_terms:GO:0016168 functional_description:Chlorophyll a/b binding protein (AHRD V1 **** Q41422_SOLTU)_contains Interpro domain(s)_IPR001344_Chlorophyll A-B binding protein ""</td><td>3</td><td>338</td><td>0.0</td><td>3</td><td>>Solyc05g008070.2.1_253</td></tr>
<tr><td>5' ATCGTGGAGATCAGTTGCGTTC 3'
| |o|o||||||||||o| |||
3' GTGCTCGTATCTCTAGTCAATGGAAGAGGAGT 5'</td><td>Solyc08g076720.2.1 genomic_reference:SL2.50ch08 gene_region:57827103-57836786 transcript_region:SL2.50ch08:57827103..57836786-go_terms:GO:0010329 functional_description:Uncharacterized ABC transporter ATP-binding protein TM_0288 (AHRD V1 *--* Y288_THEMA)_contains Interpro domain(s)_IPR003439_ABC transporter-like ""</td><td>4</td><td>2713</td><td>0.0</td><td>1</td><td>>Solyc02g036270.2.1_45</td></tr>
<tr><td>5' TACGCTTTAGCGCT-CCTGCAAC 3'
||| ||||||| || |||| |||
3' GGGCATGGGAAATCGAGAAGGACATTGGAATA 5'</td><td>Solyc10g081330.1.1 evidence_code:10F0H1E1IEG genomic_reference:SL2.50ch10 gene_region:61752173-61754621 transcript_region:SL2.50ch10:61752173..61754621-go_terms:GO:0003676,GO:0000166 functional_description:Heterogeneous nuclear ribonucleoprotein A3-like protein 2 (AHRD V1 *--- B6TV58_MAIZE)_contains Interpro domain(s)_IPR000504_RNA recognition motif RNP-1 ""</td><td>4</td><td>446</td><td>0.01</td><td>1</td><td>>Solyc02g036270.2.1_121</td></tr>
<tr><td>5' TGACAACAGTCCATGCTTTCTG 3'
|||||||||| |||||||oo
3' TAGGTGTGTTGTCAGGAACGAAAGGTATCAAC 5'</td><td>Solyc02g069450.2.1 genomic_reference:SL2.50ch02 gene_region:33915687-33919252 transcript_region:SL2.50ch02:33915687..33919252-go_terms:GO:0009538 functional_description:Photosystem I reaction center subunit III (AHRD V1 ***- Q9XQB4_PHAAU)_contains Interpro domain(s)_IPR003666_Photosystem I reaction centre protein PsaF subunit III ""</td><td>2</td><td>865</td><td>0.0</td><td>7</td><td>>Solyc05g008070.2.1_379</td></tr>
<tr><td>5' CAGTTATTGTCAAATCTGCGCA 3'
|| |o|o|o|||||| || |||
3' AGTGGTGAGTGATAGTTTACAC-CGTCAGGTA 5'</td><td>Solyc02g069450.2.1 genomic_reference:SL2.50ch02 gene_region:33915687-33919252 transcript_region:SL2.50ch02:33915687..33919252-go_terms:GO:0009538 functional_description:Photosystem I reaction center subunit III (AHRD V1 ***- Q9XQB4_PHAAU)_contains Interpro domain(s)_IPR003666_Photosystem I reaction centre protein PsaF subunit III ""</td><td>2</td><td>1752</td><td>0.0</td><td>18</td><td>>Solyc02g036270.2.1_176</td></tr>
</table>

<table>
<tr><td>5' TTTGAGAAACATGCATGGGTCA 3'
|o||||||||||| |||o|o||
3' GACGAGACTCTTTGTA-GTATCTAGACTGGGA 5'</td><td>Solyc06g035870.2.1 genomic_reference:SL2.50ch06 gene_region:21805174-21807198 transcript_region:SL2.50ch06:21805174..21807198- go_terms:GO:0035100 functional_description:Membrane-associated progesterone receptor component 1 (AHRD V1 *-*- C1GTL6_PARBA)_contains Interpro domain(s)_IPR001199_Cytochrome b5 ""</td><td>4</td><td>424</td><td>0.02</td><td>1</td><td>>Solyc05g008070.2.1_207</td></tr>
<tr><td>5' ATAAATGATCGTAGACGTGTGA 3'
|||||||||| ||o|| |oo |
3' GTTGTATTTACTAGAATTTG-ATGGTGTAAGT 5'</td><td>Solyc01g008110.2.1 genomic_reference:SL2.50ch01 gene_region:2241615-2244166 transcript_region:SL2.50ch01:2241615..2244166+ go_terms:GO:0008398 functional_description:Cytochrome P450""</td><td>2</td><td>1525</td><td>0.01</td><td>2</td><td>>Solyc05g008070.2.1_326</td></tr>
<tr><td>5' CTGTAATTTGAACGGCTCTTCT 3'
||||||o|||| |o|||| ||
3' GTGCAACATTAGACTTCCTGAGA-GAGAAAGC 5'</td><td>Solyc11g068820.1.1 evidence_code:10F0H1E1IEG genomic_reference:SL2.50ch11 gene_region:50513890-50516507 transcript_region:SL2.50ch11:50513890..50516507- go_terms:GO:0005840 functional_description:50S ribosomal protein L27 (AHRD V1 ***- Q8LEF5_ARATH)_contains Interpro domain(s)_IPR001684_Ribosomal protein L27 ""</td><td>4</td><td>507</td><td>0.05</td><td>1</td><td>>Solyc02g036270.2.1_171</td></tr>
<tr><td>5' TCTCAGCTAGTGTCGTCTTACC 3'
||| ||| || || ||||||||
3' GAAAAGA-TCGTTCTCAACAGAATGGATATGG 5'</td><td>Solyc05g008070.2.1 genomic_reference:SL2.50ch05 gene_region:2500501-2503179 transcript_region:SL2.50ch05:2500501..2503179+ go_terms:GO:0005515,GO:0000166 functional_description:Cc-nbs-lrr resistance protein""</td><td>4</td><td>555</td><td>0.03</td><td>1</td><td>>Solyc02g036270.2.1_17</td></tr>
<tr><td>5' TCAAG-AAATGAGCAATGATGAA 3'
o|||| || || ||||||||| |
3' CTAGGTTCGTTGACACGTTACTACATGTTTTA 5'</td><td>Solyc06g074820.2.1 genomic_reference:SL2.50ch06 gene_region:42747475-42749825 transcript_region:SL2.50ch06:42747475..42749825+ go_terms:GO:0015250 functional_description:Aquaporin-like protein (AHRD V1 ***- D1M6Z5_CAPAN)_contains Interpro domain(s)_IPR012269_Aquaporin ""</td><td>3</td><td>444</td><td>0.0</td><td>2</td><td>>Solyc05g008070.2.1_218</td></tr>
<tr><td>5' TACTTTGCACTGATGGTTGTGA 3'
| ||||o|||| ||||||o|||
3' ACGAAGGAAATGTGAGTACCAATACTCAATAG 5'</td><td>Solyc05g050240.2.1 genomic_reference:SL2.50ch05 gene_region:59430798-59431765 transcript_region:SL2.50ch05:59430798..59431765+ functional_description:Unknown Protein (AHRD V1)""</td><td>4</td><td>775</td><td>0.0</td><td>1</td><td>>Solyc02g036270.2.1_79</td></tr>
<tr><td>5' TGCAAGAGTTAATGGATAGGAG 3'
|o|||o||o|| ||| |||o|o
3' CAAGATGTTTTCGAT-ACCGATCTTTAAGTTA 5'</td><td>Solyc05g056070.2.1 genomic_reference:SL2.50ch05 gene_region:64606977-64608378 transcript_region:SL2.50ch05:64606977..64608378+ go_terms:GO:0016020 functional_description:Chlorophyll a-b binding protein 6A chloroplastic (AHRD V1 ***- CB11_SOLLC)_contains Interpro domain(s)_IPR001344_Chlorophyll A-B binding protein ""</td><td>2</td><td>516</td><td>0.02</td><td>10</td><td>>Solyc05g008070.2.1_312</td></tr>
<tr><td>5' TCGGAAATTGAAAATTTAGAAG 3'
||||||||||||| |o||||o
3' GCCTAGCCTTTAACTTTATAGTCTTTAGCATT 5'</td><td>Solyc02g070500.1.1 evidence_code:10F1H0E1IEG genomic_reference:SL2.50ch02 gene_region:34810672-34811115 transcript_region:SL2.50ch02:34810672..34811115+ go_terms:GO:0005515 functional_description:Susceptibility homeodomain transcription factor (Fragment) (AHRD V1 ***- Q8SAA7_ORYSA)_contains Interpro domain(s)_IPR007493_Protein of unknown function DUF538 ""</td><td>2</td><td>332</td><td>0.0</td><td>2</td><td>>Solyc02g036270.2.1_139</td></tr>
<tr><td>5' TACCTGAAATGAACCA-CTGGCC 3'
||||||| ||||| | |||o||
3' GTCACTGGACTTGACTTGATCGACTGGCTCTC 5'</td><td>Solyc11g008000.1.1 evidence_code:10F0H1E1IEG genomic_reference:SL2.50ch11 gene_region:2205263-2208572 transcript_region:SL2.50ch11:2205263..2208572- go_terms:GO:0045449 functional_description:NAC domain protein IPR003441 (AHRD V1 *-*- A9PB02_POPTR)_contains Interpro domain(s)_IPR003441_No apical meristem (NAM) protein ""</td><td>4</td><td>1344</td><td>0.03</td><td>1</td><td>>Solyc05g008070.2.1_344</td></tr>
<tr><td>5' AATGACAT-TGAGGACCATAATC 3'
|||| ||| |||oo|||||| o|
3' ACGTTACCGTATACTTTTGGTATGGGTGTGGC 5'</td><td>Solyc01g095530.2.1 genomic_reference:SL2.50ch01 gene_region:78523066-78523612 transcript_region:SL2.50ch01:78523066..78523612- functional_description:Unknown Protein (AHRD V1)""</td><td>2</td><td>230</td><td>0.03</td><td>2</td><td>>Solyc02g036270.2.1_191</td></tr>
<tr><td>5' AAAGATGTTGTGAAAGAATGCA 3'
||| ||||||||| ||||o||
3' AGGAGTTC-ACAACACTTCCTTATGTACATAT 5'</td><td>Solyc06g068560.2.1 genomic_reference:SL2.50ch06 gene_region:38881870-38886874 transcript_region:SL2.50ch06:38881870..38886874- go_terms:GO:0005215 functional_description:Folate/biopterin transporter (AHRD V1 **** B5W6N2_SPIMA)_contains Interpro domain(s)_IPR004324_Biopterin transport-related protein BT1 ""</td><td>4</td><td>1921</td><td>0.04</td><td>1</td><td>>Solyc02g036270.2.1_117</td></tr>
<tr><td>5' TTG-GCTAGTGAAGAAGGATCCT 3'
||| ||o||||||o || |||||
3' ACGAACTCGGTCACTTTATCGTAGGAGTAAAC 5'</td><td>Solyc03g007270.2.1 genomic_reference:SL2.50ch03 gene_region:1829886-1837101 transcript_region:SL2.50ch03:1829886..1837101+ go_terms:GO:0008287,GO:0003824 functional_description:Protein phosphatase 2C (AHRD V1 ***- B5A9S6_HEVBR)_contains Interpro domain(s)_IPR015655_Protein phosphatase 2C ""</td><td>4</td><td>925</td><td>0.04</td><td>1</td><td>>Solyc05g008070.2.1_365</td></tr>
<tr><td>5' TTAGAAAAGACACCTCGCGAGG 3'
|o|| |||||||| || |o||o
3' CTCGAGTCCTTTCTGTGTAG-GTTCTCGTGGT 5'</td><td>Solyc02g070980.1.1 evidence_code:10F1H1E1IEG genomic_reference:SL2.50ch02 gene_region:35092891-35093688 transcript_region:SL2.50ch02:35092891..35093688+ go_terms:GO:0005515,GO:0016168 functional_description:Chlorophyll a/b binding protein (AHRD V1 **** Q41422_SOLTU)_contains Interpro domain(s)_IPR001344_Chlorophyll A-B binding protein ""</td><td>3</td><td>338</td><td>0.01</td><td>3</td><td>>Solyc05g008070.2.1_253</td></tr>
<tr><td>5' TGCAAGAGTTAATGGATAGGAG 3'
|o|||o||o|| ||| |||o|o
3' CAAGATGTTTTCGAT-ACCGATCTTTAAGTTA 5'</td><td>Solyc05g056060.2.1 genomic_reference:SL2.50ch05 gene_region:64601085-64608155 transcript_region:SL2.50ch05:64601085..64608155+ functional_description:Ubiquitin-like protein (Fragment) (AHRD V1 *-*- A6N0B7_ORYSI)_contains Interpro domain(s)_IPR000626_Ubiquitin ""</td><td>2</td><td>2102</td><td>0.02</td><td>10</td><td>>Solyc05g008070.2.1_312</td></tr>
<tr><td>5' TAAAGATAGTTGGAAACTATTT 3'
||||| ||||| || ||||o|o
3' CCCGATTTC-ATCAAACTGTGATGAGAACTTA 5'</td><td>Solyc07g063850.2.1 genomic_reference:SL2.50ch07 gene_region:63428545-63430873 transcript_region:SL2.50ch07:63428545..63430873+ go_terms:GO:0010279 functional_description:Indole-3-acetic acid-amido synthetase GH3.8 (AHRD V1 **** B6U4E2_MAIZE)_contains Interpro domain(s)_IPR004993_GH3 auxin-responsive promoter ""</td><td>1</td><td>1852</td><td>0.0</td><td>2</td><td>>Solyc05g008070.2.1_258</td></tr>
<tr><td>5' GCTTGTTGCCTTAATGAACTGC 3'
| |||||||||| |o |||| |
3' ATCTCTAACAACGGAAGTGGTTGAGGTCCTTC 5'</td><td>Solyc07g063600.2.1 genomic_reference:SL2.50ch07 gene_region:63262431-63264147 transcript_region:SL2.50ch07:63262431..63264147- go_terms:GO:0005198 functional_description:Chlorophyll a-b binding protein 13 chloroplastic (AHRD V1 ***- CB23_SOLLC)_contains Interpro domain(s)_IPR001344_Chlorophyll A-B binding protein ""</td><td>2</td><td>711</td><td>0.02</td><td>3</td><td>>Solyc05g008070.2.1_305</td></tr>
<tr><td>5' ATTGTTGACTCCACTAATTGAA 3'
|||||| |||||| || ||||
3' AAGGTAACAA-TGAGGTTATCAACTATGTACA 5'</td><td>Solyc09g061840.2.1 genomic_reference:SL2.50ch09 gene_region:56053461-56059270 transcript_region:SL2.50ch09:56053461..56059270- go_terms:GO:0003988,GO:0003985 functional_description:3-ketoacyl CoA thiolase 1 (AHRD V1 **** C8YNG6_PETHY)_contains Interpro domain(s)_IPR002155_Thiolase ""</td><td>4</td><td>1547</td><td>0.05</td><td>1</td><td>>Solyc05g008070.2.1_215</td></tr>
</table>
