# Supplementary material for: A Short Indel-Lacking-Resistance Gene Triggers Silencing of the Photosynthetic Machinery Components Through TYLCSV-Associated Endogenous siRNAs in Tomato
Source: Front Plant Sci. 2018 Oct 11;9:1470. doi: 10.3389/fpls.2018.01470 (PMC6193080; doi:10.3389/fpls.2018.01470)
Supplement: TABLE S6 — Oligonucleotides used for quantitative reverse transcription PCR experiments of selected transcripts in TYLCSV-infected vs. mock-inoculated tissues. [file Table_6.PDF]

Supplementary Table S6. Oligonucleotides used for quantitative reverse transcription PCR experiments of selected transcripts in TYLCSV-infected vs. mock-inoculated tissues.

Transcript ID	Primer forward (5'-3')	Primer reverse (5'-3')
**UBC (SGN-U582847)**	ACCTGATGATCCACAAGATGC	GCAGCAAGTGTGGATGTTTTT
**Solyc02g063150**<br>**Solyc03g034220**	TGAGACTGAGCACGGATTTGT	AGTGCACCCAAACATAGGCA
**Solyc05g056050**<br>**Solyc05g056070**	TGGTCGTCTTGCATTGTTGG	ATGTTGTTGTGCCATGGGTC
**Solyc09g065910**	CAAGCATGTCAAGCCCAGTA	GATTGTTGCTGAGTCCAAATGG
